# Discovery of a new class of cell-penetrating peptides by novel phage display platform

**DOI:** 10.1038/s41598-024-64405-w

**Published:** 2024-06-11

**Authors:** Jinsha Liu, John Heddleston, Douglas Raymond Perkins, Jack Jia Hua Chen, Ahmadreza Ghanbarpour, Bill William Smith, Rebecca Miles, Eitaro Aihara, Sepideh Afshar

**Affiliations:** 1 Protein Engineering, Lilly Biotechnology Center, Eli Lilly and Company, San Diego, CA 92121 USA; 2 Biotechnology Research, Lilly Corporate Center, Eli Lilly and Company, Indianapolis, IN 46221 USA; 3 Genetic Medicine, Lilly Corporate Center, Eli Lilly and Company, Indianapolis, IN 46221 USA

**Keywords:** Cell-penetrating peptide (CPP), Novel phage display platform, Cytoplasmic delivery, NNJA peptides, Biotechnology, Drug discovery

## Abstract

The primary hurdles for small interference RNA (siRNA) in clinical use are targeted and cytosolic delivery. To overcome both challenges, we have established a novel platform based on phage display, called NNJA. In this approach, a lysosomal cathepsin substrate is engineered within the flexible loops of PIII, that is displaying a unique random sequence at its N-terminus. NNJA library selection targeting cell-expressed targets should yield specific peptides localized in the cytoplasm. That is because phage internalization and subsequent localization to lysosome, upon peptide binding to the cell expressed target, will result in cleavage of PIII, rendering phage non-infective. Such phage will be eliminated from the selected pool and only peptide-phage that escapes lysosomes will advance to the next round. Proof of concept studies with the NNJA library demonstrated cytosolic localization of selected peptide-phage and peptide-siRNA, confirmed through confocal microscopy. More importantly, conjugation of siHPRT to monomeric or multimeric NNJA peptides resulted in significant reduction in HPRT mRNA in various cell types without significant cytotoxicity. Sequence similarity and clustering analysis from NGS dataset provide insights into sequence composition facilitating cell penetration. NNJA platform offers a highly efficient peptide discovery engine for targeted delivery of oligonucleotides to cytosol.

## Introduction

Cell-penetrating peptides (CPPs) are versatile delivery vehicles suitable for carrying cell-impermeable therapeutic cargoes (antibodies, siRNAs, and nanoparticles) to intracellular domain^1^. First CPPs, identified three decades ago, include the truncated versions (Tat_48–60_ and Tat_49–57_) of trans-activator of transcription (Tat) protein from HIV-1 and 16-residue penetratin from *Drosophila antennapedia*^2–6^. Since then, more than 1800 CPPs have been discovered and studied^7^. In general, CPPs are cationic, amphipathic, or hydrophobic short peptides of 5–39 amino acids^8,9^ that enter the cell via direct translocation or endocytosis^10–12^. However, internalization route of a CPP should not be generalized as it depends on many factors, including biophysical and chemical properties of the peptide, CPP local concentration, cargo type, cell type, local lipid membrane composition, and experimental methodology^13^.

Great effort has been made to improve the potency of CPPs for cargo delivery. Yet the main challenge remains unresolved; CPP-cargo fusions get trapped in the endosomes upon cell entry and are eventually degraded in the lysosomes, resulting in inefficient delivery^14–16^. This limitation is reflected in the lack of clinical CPP candidates. Two phase III studies were initiated 7 years ago using Tat peptide to facilitate intracellular uptake of therapeutic peptide inhibitor targeting c-Jun N-terminal kinase (JNK)^3,5^. To date, no updates are released. The slow progress in the field might be contributed to the currently utilized methods for discovery and characterization of CPPs^13^.

Discovery of CPPs that deliver their cargos to cytoplasmic domain of the desired cell type remains the main challenge in the field^16–19^. Here, we describe a novel platform, based on phage display technology, which can overcome this challenge (Supplementary Fig. S1). Traditional selection of phage libraries against cell expressed targets comprise of addition of library to cells at 37 °C to allow binding and internalization of the receptor-bound phage. Cell lysis will result in release and identification of the entire internalized phage. Consequently, a labor and cost intensive process is required to distinguish between phage clones that are localized in the cytoplasm versus the ones trapped in the intercellular vesicles (Supplementary Fig. S2). To address this limitation, we exploited the function and structural flexibility in the loops of PIII minor phage coat protein to design a novel platform. PIII consists of three major domains, N1, N2, and C-terminal (CT), that are linked by two glycine/serine-rich (GS) linkers. Phage infection of bacteria is driven by contacts between bacterial F-pilus and TolA proteins with N1 and N2 domains of phage PIII. This facilitates phage membrane penetration, DNA insertion^20^, and hijacking of the bacterial molecular machinery to amplify phage. In addition to its critical role in infection, PIII is also widely used as the display partner for peptides, Fabs, and other proteins.

**Figure 1:**
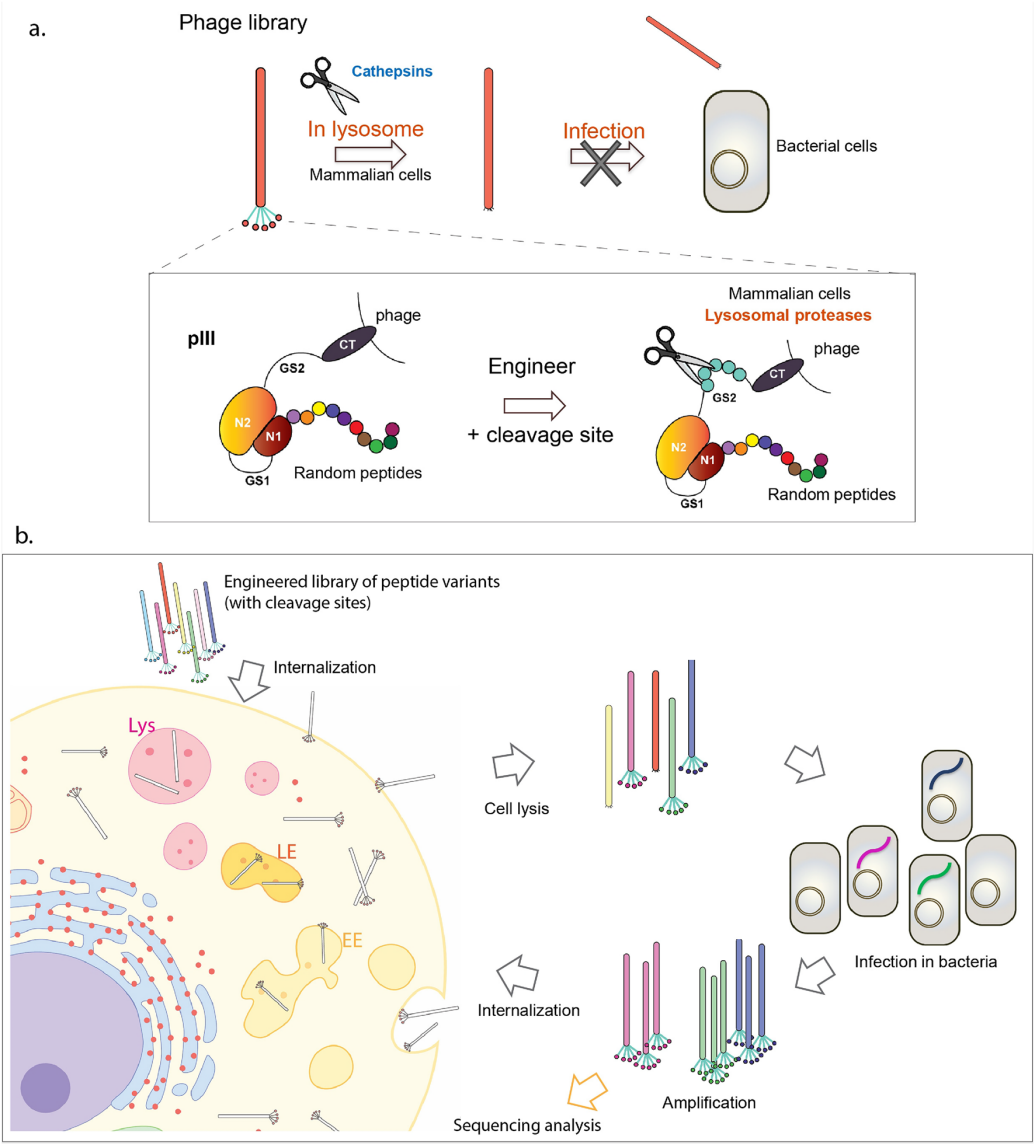
Summary of the novel phage library platform and cell-based CPP selection scheme. (**a**) Cathepsin substrate sequence is engineered in phage PIII region. Upon entering lysosome compartments led by the CPP displayed on phage, the N1 and N2 domains are removed by lysosomal cathepsin digestion, resulted in loss of infectivity in bacterial amplification step. (**b**) Overview of the cell-based internalization selection. Multiple rounds of selection are conducted to remove lysosomal localized phage clones and enrich cytoplasmic up-taken phage clones. Peptide sequences resulted in cytoplasmic localization are identified by sequencing analysis.

We merged two different biological processes to design a novel peptide phage platform called NNJA** (N**ovel peptides for **in**tracellular delivery by hi**ja**cking two cell systems) for delivery of cargos to intercellular domain of desired cell types (Fig. 1 and Supplementary Fig. S1). These processes included phage infection of bacterial cells and lysosomal protein degradation in mammalian cells by cathepsins. In short, we engineered a lysosomal peptide substrate within the GS2 linker of PIII such that phage localization to the lysosome will result in cleavage of the GS linker by cathepsins and release of N1 and N2 domains that are necessary for phage infection of bacteria (Fig. 1a). Since the resulting bald phage is unable to infect and amplify in bacteria, it will be eliminated from the library and only phage that has escaped the lysosome will advance to the next rounds of selection (Fig. 1b).

To establish a productive platform, the site at which cathepsin substrate was inserted as well as the substrate sequence was optimized. In contrast to the NNJA phage, the parental phage, lacking cathepsin substrate, was stable in the acidic pH of lysosomes and remained fully infective in the presence of lysosomal extracts. NNJA phage was used to construct a peptide phage library by displaying a random 9-mer peptide at the N-terminus of PIII. In this novel library, each phage displayed a unique peptide, and all phage contained the same cathepsin substrate with the GS2 linker of PIII. Selection of this library against three different cell lines resulted in identification of the peptides that penetrate and deliver their cargos (including siRNA) to the cytoplasmic domain. Interestingly, the identified sequences are devoid of positively charged amino acids that are present in the traditional CPPs. NNJA platform offers a unique approach for high-throughput discovery of cell-type-selective CPPs with sequences vastly different than traditional cell penetrating peptides.

## Results

### Preparation and validation of subcellular vesicles

**Figure 2:**
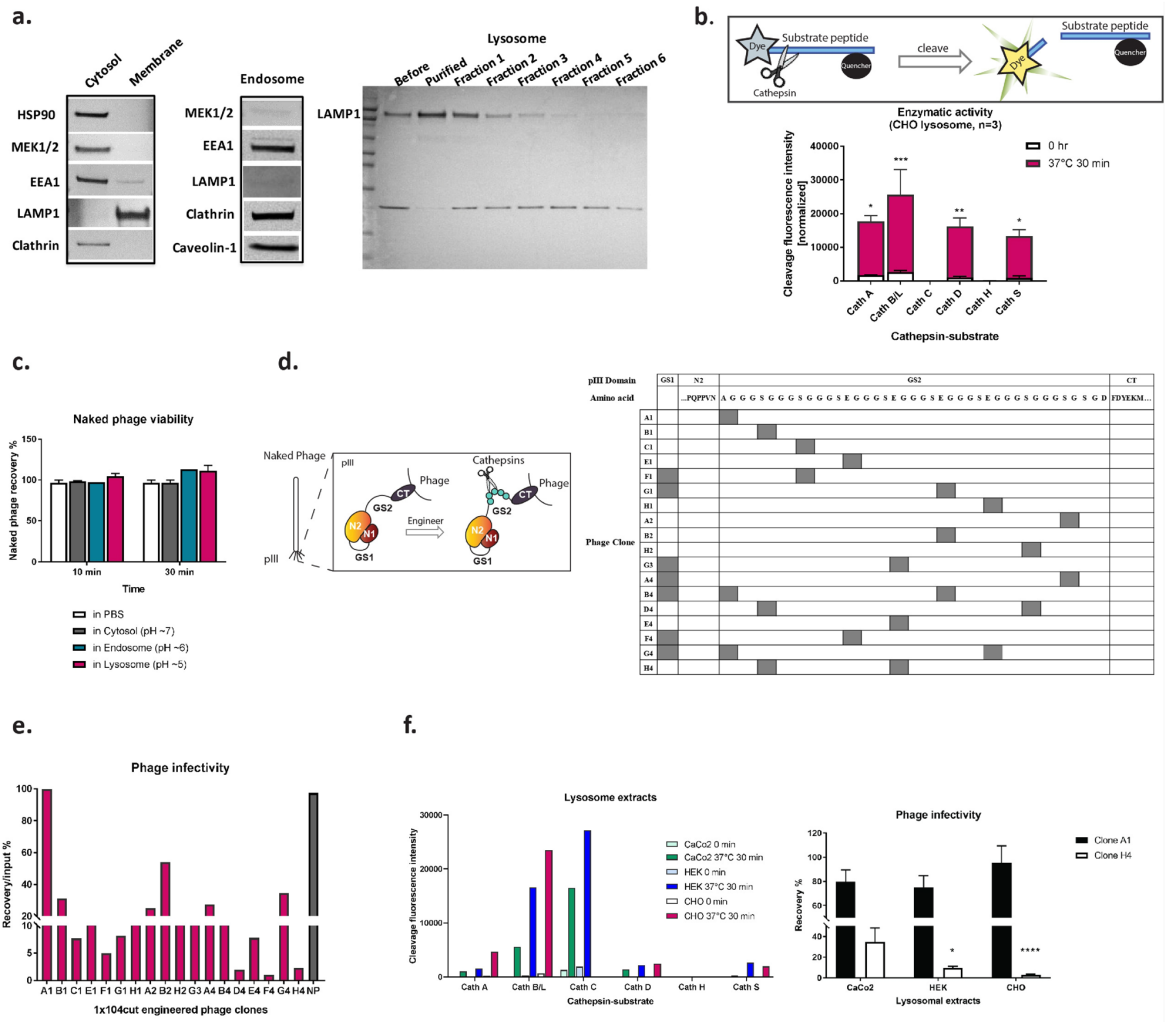
Engineering of the Cathepsin cleaving substrate in phage PIII domain. (**a**) Western blot of isolated subcellular compartment were shown by probing with subcellular markers representing cytosol, endosome, lysosome and tracking vesicles. (**b**) Fluorogenic cleavage assay was illustrated, and enzymatic activity of the major cathepsins was evaluated with specific peptide-substrate. Three individually isolated lysosomal extract from CHO cells were used. The increase in fluorescence intensity upon cleaving after 30 min incubation at 37 °C indicated that cathepsins are active from the lysosomal extract. Cath A, *p* = 0.0288; Cath B/L, *p* = 0.0001; Cath C, *ns.;* Cath D, *p* = 0.0067; Cath H, *ns.;* Cath S, *p* = 0.0326. n = 3. (**c**) Viability evaluation of naked phage in subcellular extracts. n = 2. (**d**) The design of cathepsin substrate insertion into GS linkers of PIII was illustrated. In examination of site(s) retention, eighteen unique phage clones were identified after ten rounds of overnight phage culture. The copy number and location of the substrate sequence were shown in the table. Cathepsin substrate was also inserted into GS1 linker region in seven phage clones due to the similarity of nucleotide sequences in GS1 and GS2 regions. (**e**) Representative results of phage infectivity after18 phage clones were treated with four individually isolated CHO cell lysosomal extract at pH 5. (**f**) Fluorogenic cleavage assay of cathepsin-substrate with treatment of lysosomal extracts from CaCo2, 293 and CHO cells. The infectivity rate of A1 clone and H4 clone with incubation of lysosomal extracts from three cell types. CaCo2 cells: clone A1, mean = 79.8%, SEM = 9.5%; clone H4, mean = 34.6%, SEM = 13.5%; n = 3. 293 cells: clone A1, mean = 75.1%, SEM = 9.7%; clone H4, mean = 9.5%, SEM = 1.6%, n = 3. CHO cells: clone A1, mean = 95.6%, SEM = 13.8%; clone H4, mean = 2.7%, SEM = 0.8%, n = 6. Comparison between clone A1 and H4: CaCo2, *ns.*; 293, *p* = 0.0184; CHO, *p* < 0.0001. *Cath* Cathepsin, *CT* C-terminus, *GS* glycine serine-rich linker, *NP* naked phage.

A valid subcellular fraction such as lysosome exacts, and cathepsin expression profiles within the lysosome of a particular cell type are required for an efficient cleaving of the designed substrate. We first established a reproducible in vitro subcellular fractionation procedure. For this purpose, we isolated cytosol, endosome, and lysosome components from CHO cells. Proteins specific for each compartment appeared to be enriched based on the subcellular markers detected by western blots (Fig. 2a). Cytosolic proteins, such as Heat Shock Protein 90 (HSP90) and MEK1/2, were detected in the cytosolic extracts, together with Early Endosome Antigen 1 (EEA1) and clathrin protein at a modest level, indicating the inclusion of small vesicles in the isolated cytosol fraction. Enriched EEA1, clathrin, and caveolin-1 were present in the endosomal fraction with the absence of MEK1/2 and Lysosomal-Associated Membrane Protein 1 (LAMP1). In the lysosomal extract, expectedly, LAMP1 was significantly enriched in the purified lysosomal fraction generated by the gradient ultracentrifugation.

Catalytic activity of the isolated fractions was evaluated next. Due to variable expression of lysosomal proteases in different cell types or in the same cell type under different physiological condition^21^, we chose a substrate sequence that is preferentially recognized and cleaved by seven highly expressed and universally distributed cathepsins in human cells, cathepsin A, B, C, D, H, L, and S^22,23^ for proof of concept studies. To ensure that maximum cleavage is obtained, we used the fluorogenic peptide-substrate assay to test enzymatic activities of different cathepsins. The assay was also used to quality check the lysosomal extracts from batch to batch obtained from the same cell type, as well as from different cell types. The peptide-substrates used in this study contained a fluorescent group that is efficiently quenched and is released and detected upon cleavage by cathepsins (Fig. 2b). Using this assay, a high fluorescence signal was observed from lysosomal extract compared to the readout prior to the assay onset. This suggested that cathepsin A, B/L, D and S are highly active in the tested fraction. Interestingly, no activity was observed with cathepsins C and H. This can be explained by the low levels of cathepsin C, since this enzyme is cleaved by cathepsin D at pH ~ 5, hence reducing its concentration in the low pH lysosomal fractions^24^. Low activity associated with cathepsin H can be attributed to its optimal pH, which is higher than the assay condition (i.e. pH 6)^25^. Accordingly, cathepsin H from the cytosol extract showed higher activity compared to the six cathepsins evaluated in the fluorogenic peptide-substrate (Supplementary Fig. S3). Protein expression pattern combined with functional activities of cathepsins indicated that the isolated lysosome extracts were functional and mimicking their physiological environment.

### Naked phage remain fully infective in low-pH subcellular environment in vitro

Viability of wildtype phage (naked phage) was evaluated by its addition to in vitro subcellular fractions (cytosol, endosome, and lysosome) (Fig. 2c). Post 30-min incubation of 10^9^ pfu of naked phage in 200 µl of different extracts at physiological pH, phage infectivity was assessed using *E. coli* as a host. Naked phage remained fully infective in the presence of cytosol and endosome fractions as well as in the lysosome extracts at pH 5. This indicated that parental naked phage remains viable in the presence of the lysosome fraction and that an accessible cleavage substrate within PIII is necessary to render phage unviable.

### Engineering cathepsin-substrate in phage PIII

We chose a five-residue peptide (FLVIR) as a universal substrate for the major lysosomal cathepsins. Kunkel mutagenesis was used to construct a small library within the GlySer linkers of PIII to determine the optimal substrate insertion site (Fig. 2d). Forty phage clones containing single or multiple copies of substrate were generated randomly and evaluated for stability and enzyme accessibility. To evaluate retention of the substrate within GS linker, PIII sequence was determined after ten rounds of phage amplification. As a result, 18 unique phage clones harboring one, two, or three copies of FLVIR in GS1, GS2, or both regions were identified and evaluated for infectivity after treated with cathepsins.

For this purpose, individual phage clones were incubated with CHO cell lysosomal extract. Accessibility and activity of the enzyme was assessed by determining phage infectivity post treatment. Remarkably, different trends of diminished phage infectivity were observed among the 18 phage clones tested. Naked phage, used as a control, remained fully infective (Fig. 2e). Two phage clones, A1 and H4, showed consistently high and low levels of infectivity, respectively. (A1 clone, mean = 114.8%, SEM = 19.11%; H4 clone, mean = 2%, SEM = 0.7%; Naked phage, mean = 107%, SEM = 5.9%; n = 4). The two phage clones shared the same backbone as the parental naked phage and differ only by site at which FLVIR substrate was inserted. Over 98% of H4 clone phage lost their infectivity by incubation for 30 min in the lysosome extracts indicating a robust cleaving could be achieved due to greater enzyme accessibility. In addition to the lysosomal fractions isolated from CHO cells, we evaluated infectivity of A1 and H4 phage clones in the presence of lysosomal extracts from CaCo2 and HEK-293 cell lines. Although fluorogenic cleaving assay suggested a slightly shifted cathepsin profiles in different cell types (Fig. 2f), lysosomal cathepsins recognized and cleaved FLVIR sequences in H4 phage, resulting in significant loss of its infectivity after 30-min incubation at 37 °C. We speculated that additional reduction in phage population would be achieved with multiple rounds of exposure to lysosomal cathepsins during selection.

### Selection of a novel H4_9NNK library against three cell lines

Phage clone H4 was used to construct a random nine-residue peptide library (H4_9NNK) of diversity of 7 × 10^8^ at the N-terminus of PIII. Selection was carried out with application of 10^12^ pfu of the H4_9NNK library to CHO, 293, or Caco2 cell lines. Post incubation of phage with cells for one hour at 37 °C to allow internalization, cells were thoroughly washed and stripped to remove the cell-surface binders prior to cell lysis. Upon cell entry, facilitated by the peptide displayed at the N-terminus of PIII, phage that was localized in the lysosomes would be exposed to cathepsins. As a result, PIII would be cleaved due to presence of FLVIR substrate in the GS2 linker, its N1 and N2 domains would be released, and phage would be non-infective and eliminated from the pool. Consequently, only phage clones that have entered the cell and escaped or skipped lysosomal pathway would survive and advance to next rounds of selection.

**Figure 3:**
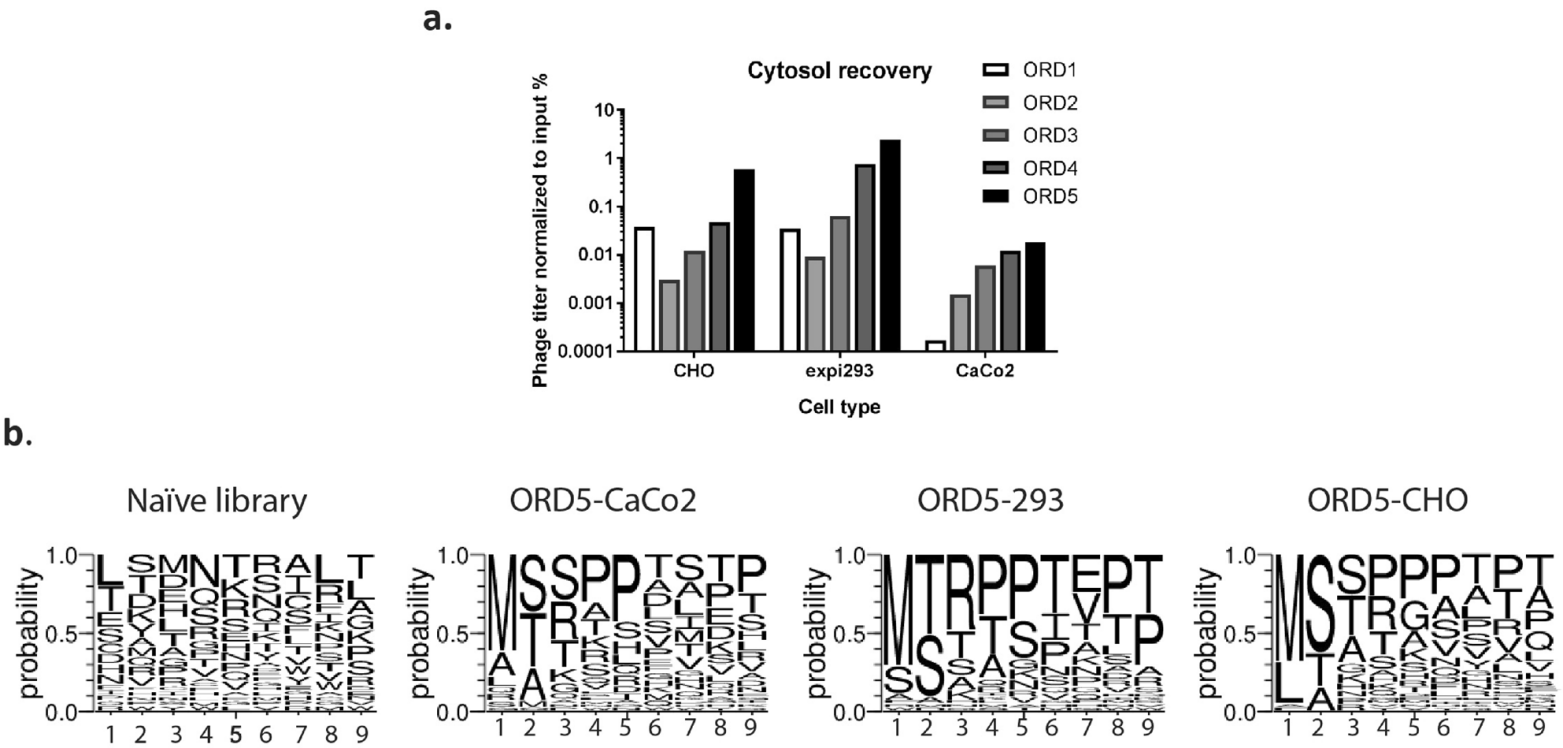
Phage titer recovery and residue enrichment after cell-based internalization selection. (**a**) Phage titers were increased steadily as the selection round progressed for three selection arms. (**b**) The frequency of twenty amino acids was analyzed at each position of the randomly picked peptide sequence identified from the naïve library prior to the five-round selection, and from the final round of selection of three cell types. Specific residues were favored after selection across three selection arms. Cell-type preferential pattern for each position was also observed. Position 1 is the N-terminus, and position 9 is the C-terminus of a peptide. ORD, output-round; input-round; A, alanine; C, cysteine; D, aspartic acid; E, glutamic acid; F, phenylalanine; G, glycine; H, histidine; I, isoleucine; K, lysine; L, leucine; M, methionine; N, asparagine; P, proline; Q, glutamine; R, arginine; S, serine; T, threonine; V, valine; W, tryptophan; Y, tyrosine.

Five rounds of selection were completed, and the selected phage was analyzed by sequencing and confocal microscopy. Improved output/input phage titer ratio as selection rounds progressed suggested enrichment of peptide sequences that localize to cell cytoplasm (Fig. 3a). Sequences of individual phage from last round of selection showed enrichment of certain amino acids at particular positions. For example, Methionine and Leucine were dominant at the first position; Serine and Threonine at second position; and Proline in the middle as well as at the C-terminus of peptides. Methionine residue was shown to play roles in lipid bilayer interaction, and support the alpha-helix conformation in polypeptides^26–29^. Proline residues provide rigidity to the peptide secondary structure, and possibly help reduce the energetic cost during membrane permeation^30^. It is important to note that in the naïve library amino acids appeared with comparable frequency at each position (Fig. 3b and Supplementary Fig. S4). Interestingly, all the selected CPPs were linear (no cysteines at any position) with high isoelectric point values and lacked repeats of positively charged residues that are common in CPPs. Another notable fact was the difference in the pattern of residue enrichment at particular positions depending on the cell type used during selection.

### Characterization of NNJA peptides as displayed on phage using confocal microscopy

**Figure 4:**
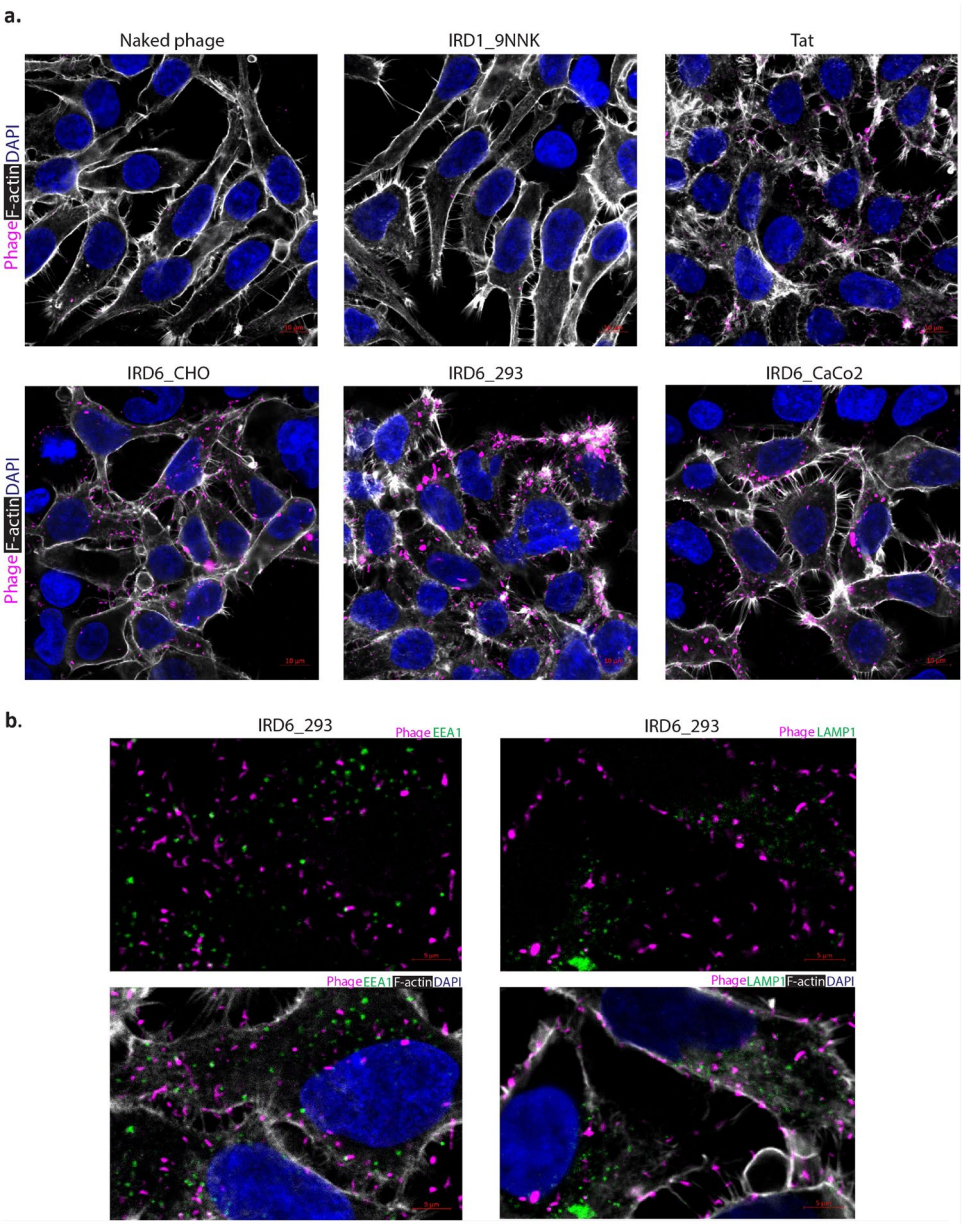
Peptide pools selected against three cell types internalized HEK cells by confocal microscopy. (**a**) The internalization of peptides in the context of phage selected after five rounds were confirmed cell-based assay by confocal microscopy. Naked phage, naïve library phage and Tat in the context of phage were used to serve as controls. (**b**) Peptides selected from expi293 cells carried phage particles internalizing in HEK293 cells by confocal microscopy. Phage was detected by anti-M13 antibody and shown in magenta. EEA1 was shown in green in the left panel, and LAMP1 was shown in green in the right panel. Filament actin was shown in white, and DAPI was shown in blue for nucleus counterstain for all the images. IRD: input round.

Confocal microscopy was used to investigate the internalization and cytoplasmic localization of the selected peptides on phage. First, we used the phage pool. The amplified phage of ORD (output round) 5 from each cell type showed significant internalization in HEK cells, detected by M13 antibody (Fig. 4a). Naked phage, naïve library (negative controls), and Tat_48–60_-peptide (positive control) displayed on phage was detected with lower signal intensity, indicating less efficient phage internalization. Importantly, we found that the internalized phage was not co-localized with EEA1 (early endosome marker) or LAMP1 (lysosome marker), suggesting cytoplasmic localization (Fig. 4b). Comparable results were observed with the same phage pool using Caco2 cells (Supplementary Fig. S5). To further characterize NNJAs, the top 37 peptides with that were highly enriched based on NGS data (Supplementary Table S1) were displayed on phage and evaluated for internalization by confocal microscopy. Some of these peptides had been identified from the selection against all three cell lines and some were cell-type selective. The result showed that internalization efficiency differed among the NNJA-phage and generally correlated with the level of their enrichment in the NGS data. Representative images showing different internalization levels of NNJA peptides on phage were shown in Supplementary Fig. S6. Confirmation of internalization for select peptides was obtained using the same approach in Caco2 cells (Supplementary Figs. S7, S8).

To investigate cell-selectivity of NNJAs, NNJA-15 on phage was evaluated by confocal microscopy in additional cell types, including N2a and SHSY5Y cells (Supplementary Fig. S9). NNJA-15 on phage was detected at a modest level by M13 antibody in the cytoplasmic domain with no co-localization with LAMP1 staining in both N2a and SHSY5Y cells. This observation suggested that NNJAs can enter cell types different than the ones they were selected against and that library depletion against un-desired cell types should be incorporated into the selection campaign to improve NNJA selectivity.

### NNJA peptides on phage deliver functional cargos to cell cytoplasm

We further evaluated the ability of NNJA peptides on phage to deliver functional groups into HEK-239 and CaCo2 cells by displaying an Avi-tag peptide on the minor coat protein pIX. Post biotinylation of Avi-tag by BirA, streptavidin-HRP was added to phage, forming a phage-protein complex (Avi[bio]/CPP-SA-HRP). We showed that NNJA peptides, Tat and penetratin displayed on phage facilitate the cellular uptake and delivery of the phage-protein complex, detected by addition of TMB to the treated cells (Supplementary Fig. S10). Internalization efficiency of NNJA peptides correlated with the level of their enrichment in NGS data. NNJAs, in general, showed comparable or superior cell entry compared to Tat or penetratin in both cell types (n = 2) under conditions tested. Negative controls, including a random peptide (sequence: TVSRELTPL) bound to streptavidin-HRP, naked phage, and streptavidin-HRP complex produced minimum signal. Cell viability was monitored by CellTiter-Glo luminescent cell viability assay and no significant alteration was observed upon addition of phage-protein complex.

Furthermore, Selected NNJA peptides (NNJA_5, 15 and 28) were inserted at various locations of an isotype antibody heavy chain (minor hinge, N-terminus and C-terminus) and showed substantial levels of internalization in SH-SY5Y cells after 3 h and 24 h incubation. The results suggested great potential of NNJA peptides in delivery antibodies into intracellular domain (Supplementary Fig. S11).

### NNJAs deliver HPRT-siRNA to cells and result in gene silencing

**Figure 5:**
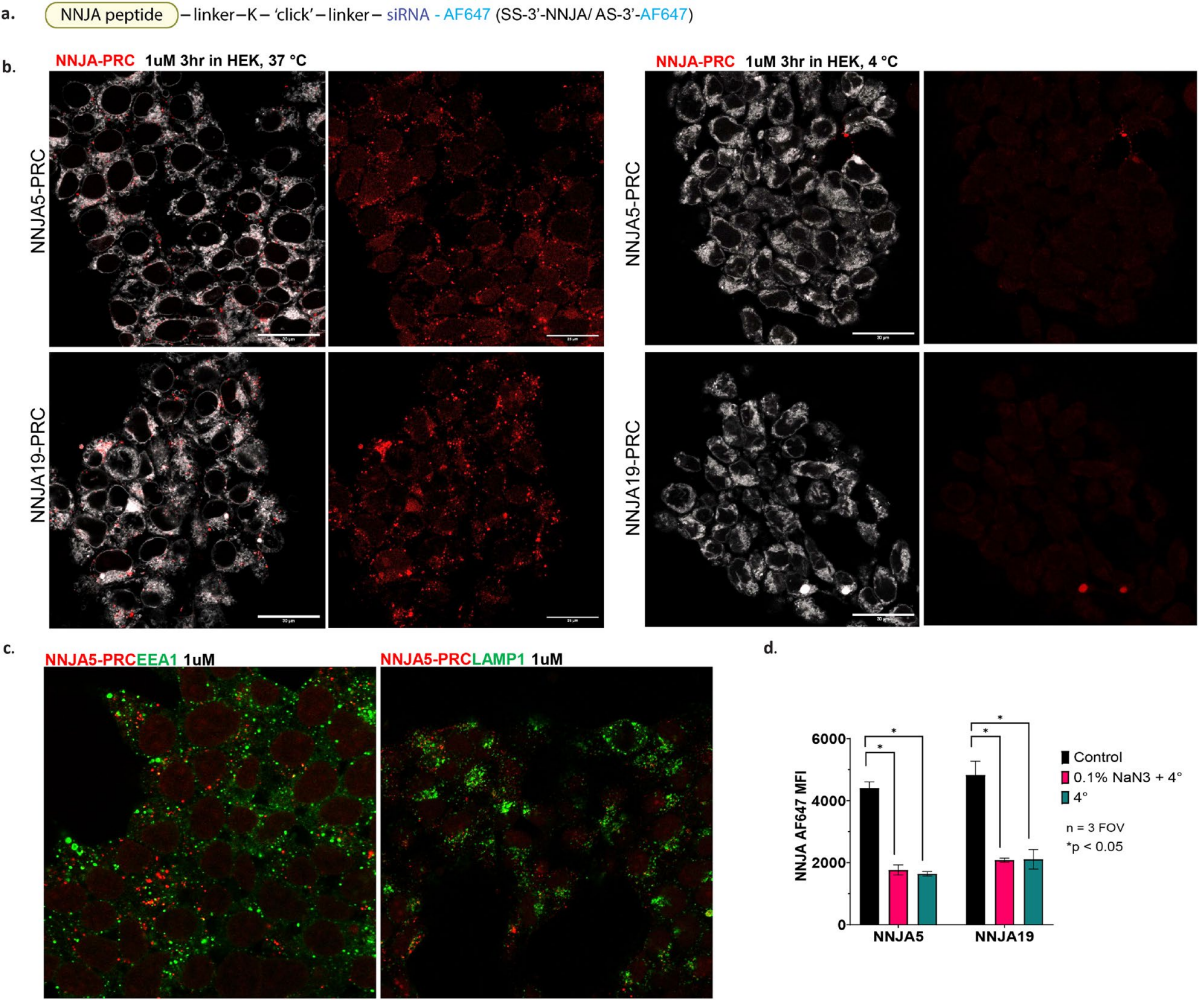
Internalization and subcellular localization of selected NNJA-siRNA-Alexa647. Two NNJA peptides, NNJA_5 and NNJA19, were directly conjugated to siRNA, with Alexa fluorophore 647 attached to the anti-sense strand of siRNA duplex (**a**). The internalization with 1 µM for 3 h was observed with both NNJA peptide RNA conjugates (shown in red), within the cell membrane (shown in white) at 37 °C, but the internalization was abolished under 4 °C treatment (**b**), and no-localization between conjugates and EEA1 or LAMP1 (shown in green) was observed under the tested condition (**c**). The internalization of the two conjugates was completely abolished under 4 °C or 4 °C with 0.1% NaN3 treatment suggesting the cellular uptake was energy dependent (**d**). *PRC* peptide-RNA conjugate, *AF* Alexa fluorophore, *AS* anti-sense strand.

The internalization and subcellular localization of two selected NNJA-siHPRT-Alexa647 were investigated by confocal microscopy. Alexa647 was labeled at the 3′ end of anti-sense strand of siRNA targeting HPRT gene (Fig. 5a). Either 1 µM of NNJA_5-siHPRT-Alexa647 or NNJA_19-siHPRT-Alexa647 conjugates were exposed to HEK cells for 3 h (Fig. 5b). Internalization was observed with both conjugates with comparable level in HEK cells at the time point tested. Further subcellular localization study revealed that no overlapping observed between NNJA_5-siHPRT-Alexa647 and EEA1 or LAMP1 markers inside the cell, indicating the conjugates are inside the cytosolic domain (Fig. 5c). The internalization of the two conjugates were then evaluated under conditions at 4 °C and 4 °C with 0.1% NaN_3_. Complete abolishment of internalization was observed for both conjugates suggesting the cellular uptake was in an energy-dependent manner, and a direct membrane entry might be the mechanisms of action for their cytoplasmic uptake (Fig. 5b,d).

**Figure 6:**
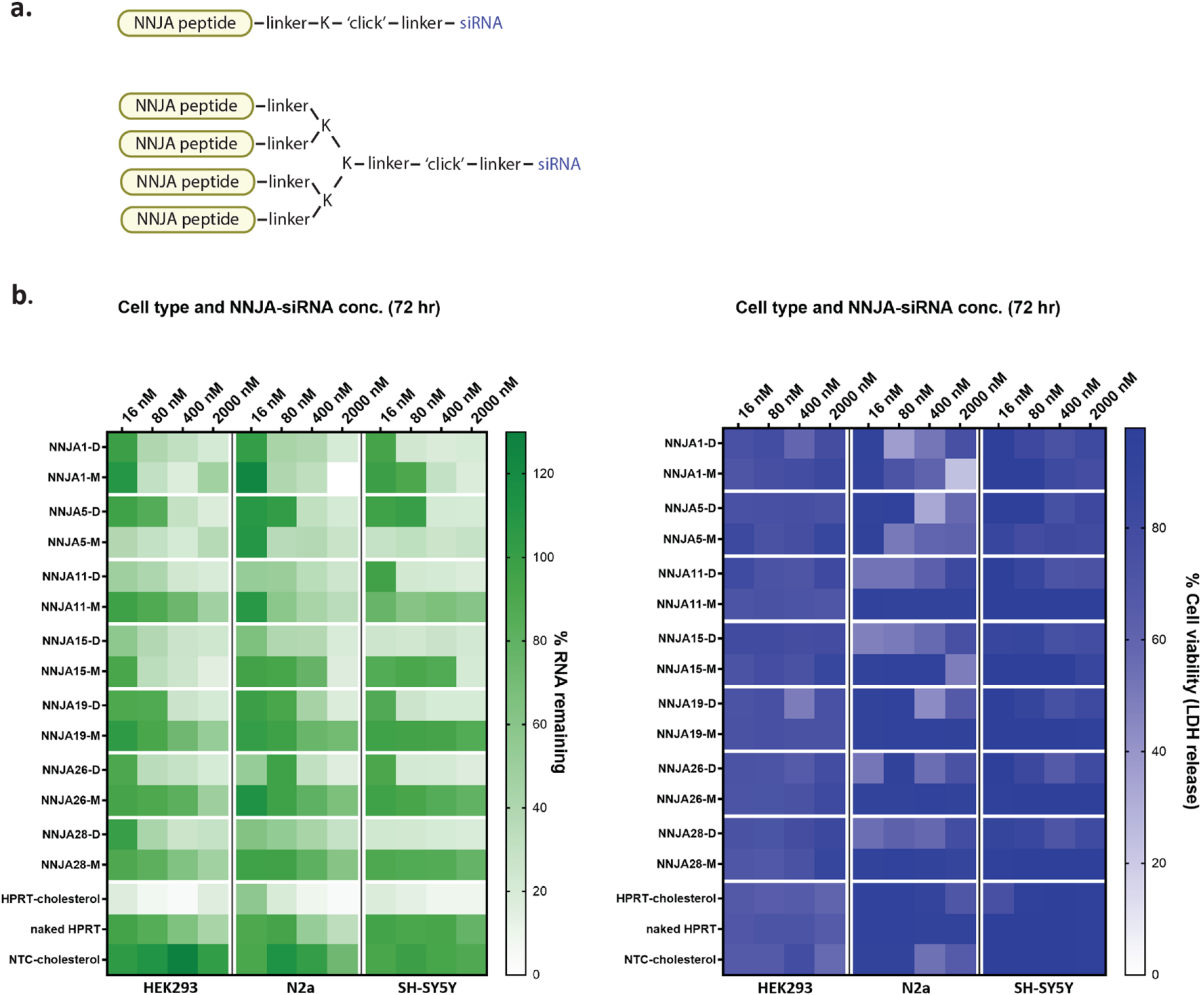
Gene silencing elicited by NNJA-siRNA delivery in various cell types. Selected NNJA peptides were synthesized in monomer and dendrimer format, and then conjugated to siRNA targeting HPRT for their self-delivery in HEK, N2a and SH-SY5Y cells (**a**). The percentage of RNA remaining inside cells was assessed by qRT-PCR at 72 h post treatment shown in the heatmap on the left. Cell viability indicated by LDH release was evaluated after compound treatment in three cell types shown on the right (**b**). *NTC* non-targeting control, *D* dendrimer, *M* monomer.

Next, we evaluate if the siRNA delivered inside cytoplasmic domain by NNJA peptides is functional. Synthetic monomeric and dendrimeric 7 NNJA peptides were directly conjugated to siHPRT with easiness of chemical reaction due to peptides’ neutral charge (Fig. 6a). Dendrimeric peptides had branched structure with four peptide copies conjugated to one siRNA to mimic peptides displayed on phage. The conjugates were tested in various cell types (e.g. HEK, N2a and SH-SY5Y cells) at different concentrations and the knockdown efficiency of HPRT gene was assessed by qRT-PCR after 72 h of treatment. Representative gene knockdown was shown as the percentage RNA remaining (Fig. 6b). HPRT-siRNA conjugated to cholesterol was used as positive control. Naked siRNA and non-targeting control (NTC) siRNA-cholesterol served as the negative controls. Overall, NNJA dendrimers showed higher siRNA reduction with a few achieving ~ 80% gene silencing and low nM IC50 values (not shown). This suggested that multivalency of the peptides enhance cell internalization, with a few exceptions. Monomeric form of NNJA_1 showed higher knockdown in 293-HEK and N2a cells compared to its dendrimer. Interestingly, the order was reversed in SH-SY5Y cells with dendrimer outperforming NNJA_1 monomer-siRNA conjugate. Another notable exception was NNJA_5 monomer that showed superior knockdown compared to its dendrimer in all cell lines tested. The cell viability, measured by lactate dehydrogenase (LDH) release assay, indicated NNJA conjugates are not toxic to cells (Fig. 6b) and do not induce significant cell death. Lower viability was observed in N2a cells with the NNJA dendrimers, however, the viability was recovered with a higher concentration of the peptide-siRNA conjugates, suggesting that LDH release assay might not be an optimal format to assess viability and that a transient LDH release can occur under certain treatment conditions.

### Bioinformatic analysis based on peptide sequence similarity

Selected NNJA peptides have demonstrated efficient cell penetration and cytoplasmic cargo delivery in various assays discussed. We further desire to understand how amino acid composition of NNJA peptides evolve over the selection rounds, and how they play roles in the cell penetration by looking into sequence similarity among all NGS data. Total of 742,034 sequences generated by NGS sampling from naïve library and output samples of 2–5 rounds selection from three cell types (total of 13 samples) were investigated (Supplementary Table S2). The proportion of unique sequences was analyzed by generating the ratio between the number of unique sequences and the total number sequences from each sample. A higher number of unique percentages indicated a more diversified sequence pool with lower level of enrichment. In all selection arms, the unique percentages decreased as the selection rounds progressed suggesting the sequence enrichment occurred through cell selection. The rate of sequence enrichment was also subjected by cell types, with a slower enrichment observed in CHO cells, and a relatively faster enrichment in Caco2 cells. Interestingly, the pattern of enrichment shown by NGS was opposite compared to the one shown as phage titer recovery (Fig. 3a), suggesting sequencing analysis was necessary to reveal details during library selection.

**Figure 7:**
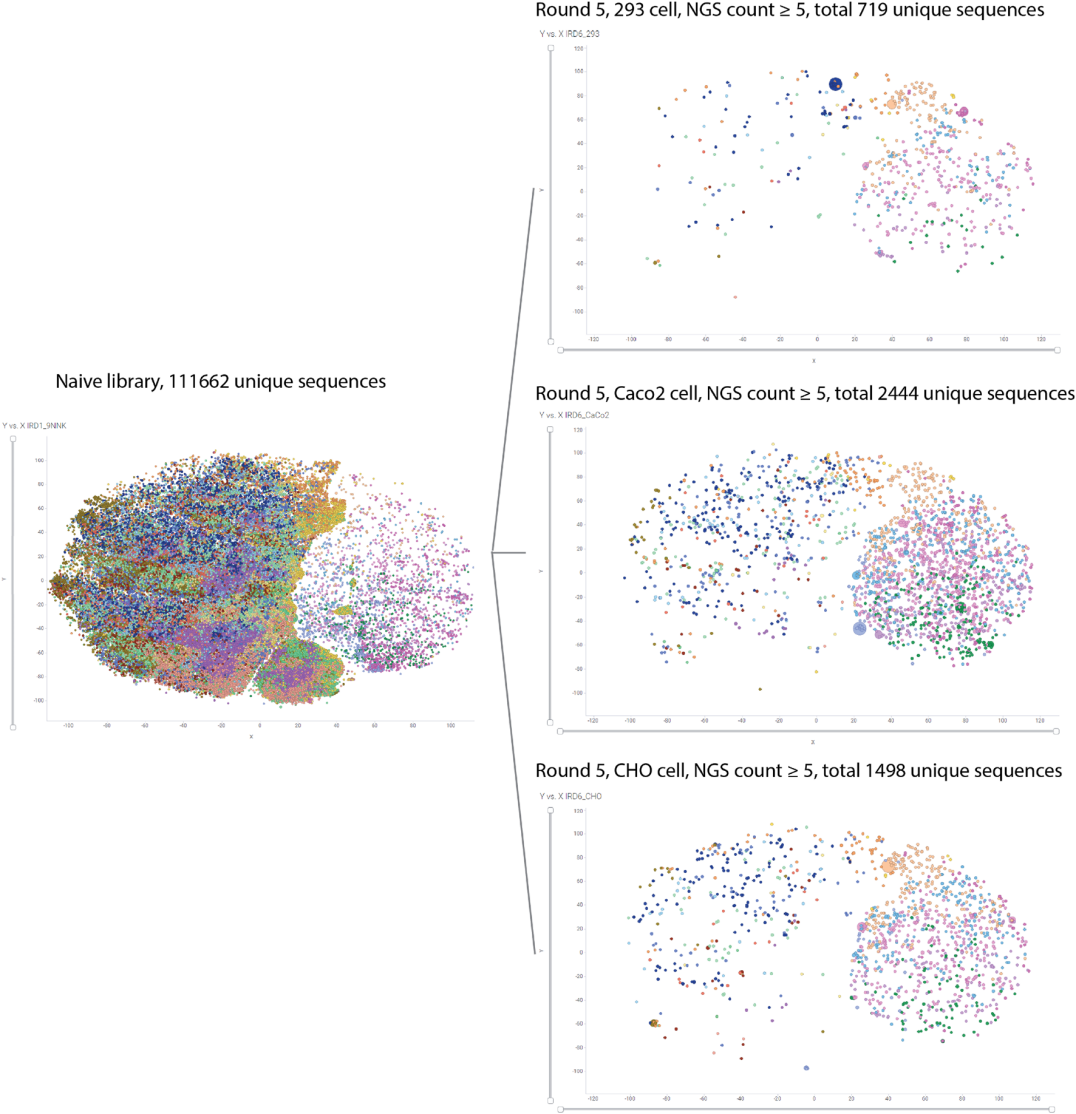
Global clustering analysis from all peptide sequences, with visualization for naïve library and peptide sequence after five rounds selection. Individual dots represent a distinct peptide sequence. Distinct colors indicate different groups of the 20 clusters. The size of a dot indicates the counts of each unique peptide from NGS: The bigger the dot, the higher counts and frequency achieved. All unique sequences were included in the naïve library clustering map over the left. Unique peptides from round 5 of each cell type from NGS were visualized in the cluster maps over the right (counts ≥ 5).

We then looked into the similarity of peptide sequences using sequence embedding tool. First, all unique sequences from naïve library and outputs as one dataset was assessed for sequence similarity. The distinct x and y coordinates as well as clustering groups were generated for every individual peptide sequence (Fig. 7 and Supplementary Fig. S12). The x and y coordinates were generated from high-dimensional sequence alignment and projected down to a two-dimensional plot. Therefore, the relationship among peptide groups is critical, but the absolute numbers of x/y axis is not correlated. All unique peptide sequences from naïve library, together with unique peptides with count ≥ 5 were visualized by TIBCO Spotfire Analyst (Fig. 7). Strikingly, about 100,000 peptides (~ 95%) located in the left hemisphere and lower corner of the naïve library clustering map were eliminated after five rounds of selection. While peptide sequences located in the right hemisphere of the naïve library map were significantly enriched after cell-based selection in all three cell types. Peptides with the highest enrichment were highlighted by their larger dot sizes in the map. Interestingly, they generally belong to different sequence clusters represented by different colors. The distribution of 37 tested NNJA peptides were also visualized in the global clustering map with the 7 NNJA peptides tested as siRNA conjugates labeled and shown in Supplementary Fig. S13.

Next, we investigated the sequence similarity within individual sample set for all 13 samples. Similar algorithm was used to generate x/y coordinates for each peptide in individual dataset, and the x/y coordinates were then generated individual plots for all 13 samples (Supplementary Fig. S14). At high-level, the shape and size of each island shown in naïve library map changed dramatically in the maps of later rounds selection of three cell types suggesting evolving changes in sequence composition. We further assigned the sequences in naïve library into 10 clusters based on sequence similarity, and tried to decipher what were the characteristics of each cluster pool. Note that x/y coordinates of the same peptide sequence would be shown differently in another dataset because it was embedded in a different sequence pool for similarity assessment. We tracked if sequences in each of the 10 clusters prior to selection were further enriched or eliminated due to cell-based selection by cell type (Supplementary Figs. S15–S17). Note that cluster 4 was divided into two groups (4–1 and 4–2) as it presented clear separations with distinct sequence signatures in the naïve library map. The level of enrichment or diminishment from each of 10 clusters from naïve library was summarized in Table 1, together with peptide properties including hydrophobicity, isoelectric point, and charges. Clear patterns in sequence composition were reflected. First, peptide sequences containing Cysteine residues in cluster 1 and 7 were almost if not completely eliminated from the selection in all cell types. The diminishment of Cysteines in sequences suggested that disulfide bonds were not favored or needed for the process of cytoplasmic entering. Second, peptide sequences containing multiple positive charge amino acids, such as from cluster 3 and cluster 4–2 had minimal presence after the selection. Third, peptide sequences started with Methionine at N-terminus (cluster 4–1) were significantly enriched and further expanded. The cluster of N-term Methionine variants became the majority population for all three cell types indicating critical role of Methionine and the particular sequence position. Note the sequences from cluster 4–1, 9 and 10 that were highly enriched after selection, and they were more hydrophobic and neutral in charge in comparison to the classic CPP listed in Table 1, suggesting NNJA peptides might utilize different mechanisms of action to interact with cell membrane and elicit more efficient cell penetration. An alternative view in Supplementary Fig. S18 showed the trends of diminishment of Cysteine containing sequences and enrichment and expansion of N-term methionine containing sequences throughout the selection. We traced the evolution of Methionine containing sequences at non-N-terminus positions and found that they presented in a minor population after 5 round selection.

**Table 1:** Peptide properties from 10 clusters of naïve library and sequence enrichment at cell-based selection completion. The most commonly used classic CPP sequences were included for direct comparison.

Aminos acid composition signatures in naïve library					Enrichment after 5 rounds cell selection		
Cluster group	Average hydrophobicity (KyteDoolittle scale)	Average isoelectric point (EMBOSS)	Average charge at pH 7.4 (Lehninger)	AA highlight	293 cells	Caco2 cells	CHO cells
Cluster 1	0.24	7.23	0.26	Cys containing	Eliminated	Minimum	Eliminated
Cluster 2	− 0.77	7.31	0.13		High	Slight	Slight
Cluster 3	− 1.31	10.42	1.71	Multiple positive charged AA containing	Eliminated	Minimum	Minimum
Cluster 4–1	− 0.34	8.42	0.65	N-term Met	High	High	High
Cluster 4–2	− 1.08	11.05	1.99	Multiple positive charged AA containing, internal-Met containing, Pro containing	Minimum	Minimum	Minimum
Cluster 5	0.18	7.32	0.12		Minimum	Slight	Modest
Cluster 6	− 1.31	10.86	1.69		Modest	Minimum	Minimum
Cluster 7	− 0.62	8.38	0.93	Cys containing	Eliminated	Eliminated	Eliminated
Cluster 8	0.10	8.83	0.79		Eliminated	Minimum	Minimum
Cluster 9	− 0.84	7.36	0.11		Modest	Modest	High
Cluster 10	− 0.09	7.59	0.25		High	Slight	High

Classic CPP	Amino acid composition signatures						
	hydrophobicity (KyteDoolittle scale)	Isoelectric point (EMBOSS)	Charge at pH 7.4 (Lehninger)	AA sequence			
Tat48–60	− 3.7	13.2	8.0	GRKKRRQRRRPQ			
Penetratin	− 1.7	12.8	7.0	RQIKIWFQNRRMKWKK			
9R	− 4.5	13.4	9.0	RRRRRRRRR			
8K	− 3.9	11.6	8.0	KKKKKKKK			

*CPP* cell-penetrating peptides, *AA* amino acid, *Cys* cysteine, *Met* methionine, *Pro* proline.

## Discussion

In the current study, an effective and novel phage display platform was established to select CPPs. As a proof of concept, we have applied the novel phage library to screen against various cell types, including CHO, HEK293, and CaCo2 cells. No engineering process to the target mammalian cells was required. The new class of CPPs discovered in this study was remarkably distinguishable in size and biophysical properties from the conventional CPPs^31^. The CPPs we discovered were short with nine residues in length presenting specific secondary structures. They also processed high isoelectric point (PI) value with minimum presence of positive charged residues. In addition, specific residues were enriched at a particular position of the peptide sequences, such as Methionine, Leucine, Serine, and Proline. Specific uptake mechanisms of action facilitated by such residue enrichment need to be further studied, for example by Alanine scan, Methionine mutation to oxidized Methionine or oxidized resistant analogue (i.e. Norleucine) of the peptide sequence.

Superior penetration rate and level was observed with some NNJA peptides in the context of phage, compared to Tat and penetratin peptides in multiple cell-based assays. NNJA peptides also demonstrated great potential in delivery larger molecules such as antibody as peptide-antibody fusion into the intracellular domain. The fusion protein by inserting NNJA peptides at various location of antibody provides the flexibility of incorporating peptide multi-valency, possibility of engaging different targets, and straightforward protein generation through plasmid cloning, expression and purification processes. The delivery of isotype antibody by NNJA peptides also expanded the possibility of cargo types, such as to delivery larger proteins and enzymes (i.e. CRISPR-Cas9) for functional targeting^32^.

As synthetic peptides, NNJA were proven to deliver siRNA targeting HPRT mRNA into the cytoplasm domain leading to significant gene silencing in multiple cell types in an energy required manner. Depending on the NNJA peptide sequence, monomer and dendrimer peptide siRNA conjugates generated various levels of the gene knockdown with some level of cell type preference. Interestingly, NNJA_19 monomer siRNA conjugate showed weaker gene silencing in HEK cells after 72 h treatment, compared to NNJA_5 monomer siRNA conjugate, despite of the comparable level of internalization at 3 h observed by confocal imaging. This result indicates the difference in uptake kinetics between different NNJA siRNA conjugates, and detailed mechanisms of action of individual NNJA-siRNA need to be further deciphered.

Thousands of peptide sequences were generated after library selection, and it is challenging to chemically synthesize a large number as pure peptides and to link to various cargos by either chemical conjugation or as fusion for intracellular delivery assessment. Commonly, we would pick and sample a limited number of sequences for chemical synthesis and conjugation. In our case, we only selected total of 7 NNJA peptides outside the context of phage, and evaluated them as peptide-antibody fusion or peptide-siRNA conjugates in cell-based assays. In order to look into a larger number of sequences and understand the amino acid composition, we developed a sequence similarity analysis and clustering using sequence-embeddings generated from a protein language model. The analysis allowed us to take all 742,034 sequences generated by NGS from sampling naïve library and all outputs (13 dataset total) and looked at the sequence similarity as one dataset or as individuals. Remarkable patterns were revealed that Cysteine containing sequences were eliminated indicating disulfide bonds were not favored in cytoplasmic entry; N-terminus Methionine containing sequences were significantly enriched and expanded possibly for membrane interaction. Proline residues were preferred in the sequences for potential rigidity of peptide structures. Particular clusters from naïve library were emphasized in enrichment during selection, and more sampling from those clusters could be beneficial in searching for more potent peptides by testing in cell-based assays. Furthermore, the experimental data could be fed back to a machine learning algorithm, and to develop a prediction tool of peptide sequences for efficient cell penetration.

In summary, the NNJA platform can be very powerful to generate CPPs for specific delivery purposes. First of all, the substrate sequence inserted into phage library can be tailored based on the cathepsin profiling within the cell type of interest, to achieve higher level of clearance for lysosomal localized peptide-phage. For example, cathepsin S is highly expressed in T cells, and substrate sequence cleaved by cathepsin S can facilitate more efficient library selection in the cell-based assay. Second, selection and/or counter-selection with the novel phage library can be performed against a variety of cell types to select cell/tissue-type specific CPPs for a particular therapeutic application. Third, the NNJA platform can enhance targeted delivery (i.e. receptor-mediated uptake) by identifying peptide sequences that facilitate endo-lysosome release through receptor-mediated endocytosis pathway (Supplementary Fig. S19). Finally, the phage library can be screened against ex vivo models, such as organoid system, as well as in vivo models to select peptides that can cross the cell or tissue barrier by transcytosis, such as for oral delivery purposes.

## Methods

### Cells and reagents

Chinese hamster ovary (CHO-2F9 gifted from protein biosciences group/Eli Lilly, abbreviated as CHO) cells were grown in suspension with medium prepared in-house (M9195 + 12 mM l-glutamine). Expi293 (abbreviated as 293) (Life technologies) cells are also suspension cells maintained in culture medium (Cat. No. A14351-01, Gibco) in 8% CO_2_ at 37 °C. Adherent Colon carcinoma (CaCo2) cells were cultured in Dulbecco’s Modified Eagle’s Medium (DMEM) supplemented with l-glutamine, 10% heat-inactivated (HI) FBS, 1 mM sodium pyruvate and 25 mM HEPES. Adherent HEK293 (abbreviated as HEK) cells were grown in Minimum Essential Medium (MEM) supplemented with 10% HI FBS, 1 × non-essential amino acids, 1 mM sodium pyruvate, and 0.075% sodium bicarbonate, and used for microscopy imaging and cytotoxicity assays. Neuro2a (N2a) cells are cultured in DMEM supplemented with 10% HI FBS. SH-SY5Y cells are grown in Eagle’s minimal essential medium (EMEM)/Ham’s F12 (1:1), supplemented with 10% HI FBS. If not specified, cells were incubated in 5% CO_2_ at 37 °C.

### Antibodies

Anti-caveolin-1, Cat. No. 3267S, Cell signaling; anti-clathrin, Cat. No. 4796S, Cell signaling; anti-EEA1, Cat. No. ab2900, Abcam; anti-HSP90, Cat. No. 8165S, Cell signaling; anti-LAMP1, Cat. No. ab24170, Abcam; anti-MEK1/2, Cat. No. 8727S, cell signaling. Secondary antibodies: goat anti-mouse (HRP) and goat anti-rabbit (HRP) from Jackson Lab. For confocal imaging, anti-M13-Alexa 647 (in-house), anti-LAMP1, Cat. No. 9091, Cell Signaling; anti-F-actin-DyLight488, Cat. No. PI21833, ThermoFisher; DAPI from Invitrogen; Alexa Fluor 488, Alexa Fluor 568, and Alexa Fluor 647-coupled fluorescent secondary antibodies from Life Technologies.

### Western blot

Ten μg/lane of the protein were separated by 4–12% SDS-polyacrylamide gel electrophoresis (PAGE) and transferred to polyvinylidene difluoride (PVDF) membranes. Blots were blocked in Casein for 1 h at room temperature. Subcellular markers were detected using specific antibodies and visualized after incubation with horseradish peroxidase (HRP)-conjugated secondary antibodies using the TMB blotting detection.

### Subcellular fractionation

Cytosolic and endosomal extraction were prepared according to the procedures suggested by the manufactures from ThermoFisher Scientific (Cat. No. 89842) and Invent Biotechnologies (Cat. No. ED-028) respectively. The starting cell number is 5 × 10^6^ cells for one cytosolic extraction, and 3 × 10^7^ cells for one endosome extraction. Lysosomal isolation from different cell types were optimized based on Abcam kit (Cat. No. ab234047) for homogenization step and increased isolation scale. The starting cell number for one lysosomal isolation is 2 × 10^8^ cells.

### Cathepsins enzymatic cleavage assay

Six fluorogenic peptide substrates were purchased from R&D Systems, Bachem, and Chemimpex. Cathepsin B and L share the same fluorogenic peptide-substrate, and the rest five cathepsins cleave at a specific fluorogenic peptide-substrate. The peptide substrate for each cathepsin is as follows: cathepsin A (Cat. No. ES005, R&D Systems), cathepsin B/L (Cat. No. ES008, R&D Systems), cathepsin C (Cat. No. I-1215, Bachem), cathepsin D (Cat. No. ES001, R&D Systems), cathepsin H (Cat. No. 05859, Chemimpex), and cathepsin S (Cat. No. ES002, R&D Systems). Five µl of 200 µM peptide substrate was incubated with 5 µl of lysosomal extraction in citrate buffer (pH 5) for 30 min at 37 °C. Fluorescence emission level of each peptide substrate was normalized by subtracting the background fluoresce generated by the peptide substrate only in citrate buffer. Higher fluorescence signal detected indicates higher level of enzymatic activity of a particular cathepsin from the lysosome enrichment.

### Engineering cathepsin-cleavable substrates into GS1 and/or GS2 linker domain of phage PIII

Phage clones with cleavable substrate(s) were generated using the previously described M13 IX104 bacteriophage vector^33^. *Escherichia coli* (*E. coli*) strain RZ1032 (ATCC 39737), which lacks functional dUTPase and uracil glycosylase, was used to prepare uracil containing single-stranded DNA (du-ssDNA) of the IX104 vector. In *E. coli*, dUTPase and uracil-N glycosylase serve to play roles in DNA repair and ensure fidelity of DNA replication by removing any uracil incorporated into the bacterial genome^34^. Oligonucleotide sequences encoding the five-residue FLVIR sequence were designed, and the corresponding reverse complement oligo was annealed to various location in PIII GS2 linker region of du-ssDNA IX104 vector by Kunkel mutagenesis. Electrocompetent *E*. *coli* DH10B cells were used for transformations. Forty unique phage clones were confirmed by Sanger sequencing. Ten rounds of overnight phage culture were grown to evaluate substrate sequence retention for each phage clone. Sanger sequencing was performed after each round of phage culture to confirm the substrate insertion. Final phage clones with the retained substrate insertion were then evaluated for cathepsin accessibility with lysosomal extract from different mammalian cells.

### Phage display library construction and CPPs selection

With the backbone structure of phage clone H4 with the Cathepsin cleavable substrate insertion in GS2 linker, a nine-residue library of oligonucleotides (9NNK) encoding random amino acid sequences was designed such that the random NNK region was flanked by nucleotides complementary to the vector. The 5′-phosphorylated reverse complement oligo was annealed to dUssDNA 8 + 11 vector using^33,35^ Kunkel mutagenesis and extended to form double stranded DNA (dsDNA)^36^. Electrocompetent *E*. *coli* DH10B cells were used for transformations. A pool of transformants was titered to determine the diversity of the library. Phage were then amplified in the presence of freshly grown XL-1blue cells overnight on LB plates at 37 °C. The next day, phage was eluted off the plate, precipitated, titered and stored at -80°C in the presence of 50% glycerol until use.

Before applying the engineered phage library to mammalian cells, complete culture medium was replaced with serum-free culture medium, and cells were incubated for 1 h at 37 °C. For primary selection, 10^12^ phage from the library were incubated with 10^7^ of various cultured cells as different selection arms, for 1 h at 37 °C inside a tissue culture incubator (on rotator for suspension cells). After internalization, cells were gently washed with cold phosphate buffer saline (PBS) once and followed by cold low-pH stripping buffer (culture medium adjusted to pH 2.5 for CHO cells; 100 mM glycine, 150 mM NaCl, pH 2.5 for 293 and CaCo2 cells) for 5 min twice. Cells were immediately washed with cold PBS for three times. Cells in suspension were spined down at 300×*g* for 5 min at 4 °C, whereas adherent cells were scraped on ice and proceeded directly to the next step. Washed cells were gently lysate using the cytosolic extract reagents (ThermoFisher Scientific) to collect phage particles in about 1.5 ml volume. The recovered phage from cytosolic region were amplified by plating with 5 ml of freshly grown mid-log XL-1blue cells with 40 ml top agar onto large LB plates (Cat. No. L6100, Teknova). Plates were incubated overnight at 37 °C. On the second day, phage were eluted by incubation with 30 ml phage suspension buffer (100 mM NaCl, 8.1 mM MgCl_2_, 50 mM Tris–HCl, pH 7.5) for 2 h at room temperature. Then the plate surface was gently scraped, and the phage elution is collected. The eluted phage samples were spined, precipitated, and titered for use in subsequent rounds of selection. Five rounds of selection were conducted. Starting from the output of round three (ORD3) to the completion of the whole selection, phage plaques were random-picked, eluted, PCR-amplified, and sequenced by Sanger sequencing. The amplified phage samples of ORD 3–5, serving as the input rounds (IRD) 4–6 were analyzed by Next Generation Sequencing (NGS) to identify peptide sequences and their occurring frequencies.

### Sanger sequencing

Amplicons were first purified using Exonuclease I and Fast AP. The purified PCR product was used as the DNA template for the Big Dye Terminator 3.1 cycle sequencing chemistry. The sequencing reaction was then purified with Seq DTR magbind beads and loaded onto a Bioanalyzer 3730XL for sequencing by capillary electrophoresis.

### Next generation sequencing (NGS)

Only a small fraction of the output phage samples was sequenced by NGS. Amplicons went through a two-step PCR process. The first PCR was the addition of the SBS sites for Illumina’s sequencing primer and the second PCR was the addition of the Nextera Indexes to allow for sample demultiplexing. Both PCR steps were purified using a 1.8 × ratio of MagBind RxnPurePlus beads. The purified 2nd PCR products were then quantified by qPCR using a ViiA 7 and a Bioanalyzer 2100 fragment analyzer. The samples were then pooled in equal molar ratios and denatured following Illumina’s MiSeq System Denature and Dilute guide. Samples were loaded on the MiSeq at a concentration of 12.5 pM and 20% PhiX was spiked in. The run conditions for the MiSeq were a single direction of 130 cycles and 1 M reads via V2 Nano Reagent Kit. Amino acid sequences encoded by nucleotides were then trimmed and cleaned based on the primer sequences and linker sequences. Counts and frequencies of each amino acid sequence was reported.

### Immunocytochemistry and confocal microscopy

Cells were fixed with 4% paraformaldehyde (PFA) on ice for 20 min after the washing steps. Fixed cells were washed with PBS for three times to remove any excess PFA, and followed by 1 h blocking in 3% bovine serum albumin (BSA) in Tris buffer saline (TBS) with 0.2% triton X-100, and antibodies staining in TBS with 0.2% triton X-100. Primary antibodies were incubated with cell samples overnight at 4 °C. Following PBS washes, species-specific Alexa Fluor 488, Alexa Fluor 568, and/or Alexa Fluor 647-coupled secondary antibodies were used for signal detection. Imaging data collection was conducted on an LSM800 (Zeiss) confocal microscope, using a 40× (NA 1.1 W LD C-Apochromat, Zeiss) objective. Controls treated with secondary antibody were shown negative or undetectable signal.

### Double-displayed phage carrying horseradish peroxidase (HRP) in cell-based assay

Avi-tag sequence (GLNDIFEAQKIEWHE) was displayed on the N-terminus of phage minor coat protein pIX. At the same time, CPP (e.g. NNJA, Tat or penetratin peptide) was displayed on PIII of the same phage clone, by inserting the corresponding nucleotides into 1X104 phage backbone by Kunkel mutagenesis. A randomly picked peptide (TVSRELTPL) from the naïve library was incorporated in the setting and served as one of the negative controls. The method was described previously^37^. Cell viability was evaluated in a replicated experimental group under the same assay condition using CellTiter-Glo luminescent cell viability assay from Promega.

### NNJA peptide–antibody fusion internalization in SH-SY5Y cells

Selected NNJA peptides (NNJA_5, 15 and 28) were inserted into different locations of heavy chain of an engineered isotype IgG4, including minor hinge (MH), N-terminus (Nterm) and C-terminus (Cterm) by cloning and CHO cell expression. Among all the expressed combinations of peptide and inserted locations, NNJA_5-Nterm and NNJA_28-Nterm were not evaluated in cell assay due to incorrect molecular weight of the molecules. The engineered IgG4 scaffold contains a free Cysteine residue in the CH1 region of heavy chain. Alexa fluorophore 488 (Cat. No. A10254) was labeled onto the free Cysteine using maleimide-thiol reaction, and DAR = 2 dye per peptide-antibody fusion was confirmed by MALDI-MS. Peptide-antibody fusions at 100 nM were assessed for their internalization levels in SH-SY5Y cells by incubation for 3 h and 24 h with base media supplemented with 100 µg/ml goat gamma globulin. Cells were treated with 0.25% trypsin EDTA for 15 min to remove surface-bound molecules and read by flow cytometer after washing in FACS buffer. CPP10 with AF488 fluorophore was used as the control. CPP10 was CPP-12 as original name in the published study^38^. Results were analyzed on single non-debris events, and cells only control was used to set as the negative gate.

### Peptide synthesis and conjugation

Synthetic peptides were ordered from CPC scientific with > 95% purity. NNJA peptides in the formats of monomer or dendrimer were conjugated by click chemistry to siRNA targeting hypoxanthine–guanine phosphoribosyltransferase (HPRT) gene (designed in-house, synthesized from Biosynthesis) at the C-terminal end of the peptide. For the confocal microscopy visualization, Alexa fluorophore 647 was added to the 3′ of antisense of siRNA that then formed duplex by annealing to the sense strand siRNA which was conjugated to NNJA peptide at 3′ end.

### NNJA-siRNA knockdown assay

Ten thousand cells, such as HEK, N2a and SH-SY5Y cells were plated in Accell media followed by the treatment of the compounds (siRNA controls or NNJA-siRNA). The concentration of compounds started at 2 µM followed by 1:5 dilutions. Cells were then incubated at 37 °C, with 5% CO_2_ for 72 h. The knock-down efficiency achieved by NNJA-siRNA was assessed by qRT-PCR using Cells to Ct followed by TaqMan (Cat. No. A25603, ThermoFisher) with HPRT primer/probes. Cell viability was evaluated by CytoTox 96 Non-Radioactive Cytotoxicity Assay (Cat. No. G1780, Promega). Statistical analysis was generated in Prism using 3-parameter curve fit.

### Statistics

Statistical analysis was conducted using standard error of the mean (SEM), two-way ANOVA and multiple comparison test on GraphPad Prism (version 9.1.1), unless otherwise stated. Statistical results (e.g. *p* value) are described in figure legends and use confidence intervals of 95%.

### Sequence clustering using protein language model embeddings

A protein language model for similarity analysis and clustering of the peptide sequences was used for sequence embeddings. The sequences embeddings for every sequence from naïve library and all rounds were clustered (global clustering) using ESM-2 language model with 650 M parameters^39^. Next, the sequences were clustered by their embeddings similarity as follows: For faster and more efficient clustering, the sequence embeddings dimensions for each sequence were reduced to twenty from 1280 using principal component analysis (PCA) and clustered into 20 groups using Gaussian mixture models (GMM) algorithm. For sequence visualization in a 2D plot, the sequence dimensions were reduced to two dimensions using the T-SNE algorithm^40^. For sequence clustering generated for individual sample from either naïve library or each round, similar analysis was conducted with 10 clusters per sample. The peptide sequences, NGS counts, and the x/y coordinates generated from the sequence clustering were visualized by TIBCO Spotfire Analyst (version 12.0.2). Peptide properties such as hydrophobicity (KyteDoolittle scale), isoelectric point (PI) (EMBOSS) and charge (Lehninger) were calculated by peptides.py^41^.

### Supplementary Information

Supplementary Information.

## Data Availability

All data generated or analysed during this study are included in this published article, its Supplementary Information file and patent WO2023/159105 A1.
