# Supplementary material for: Discovery of a new class of cell-penetrating peptides by novel phage display platform
Source: Sci Rep. 2024 Jun 11;14:13437. doi: 10.1038/s41598-024-64405-w (PMC11167021; doi:10.1038/s41598-024-64405-w)
Supplement: Supplementary file 1 — Supplementary Information. [file 41598_2024_64405_MOESM1_ESM.docx]

**Supplementary Figures and tables.**


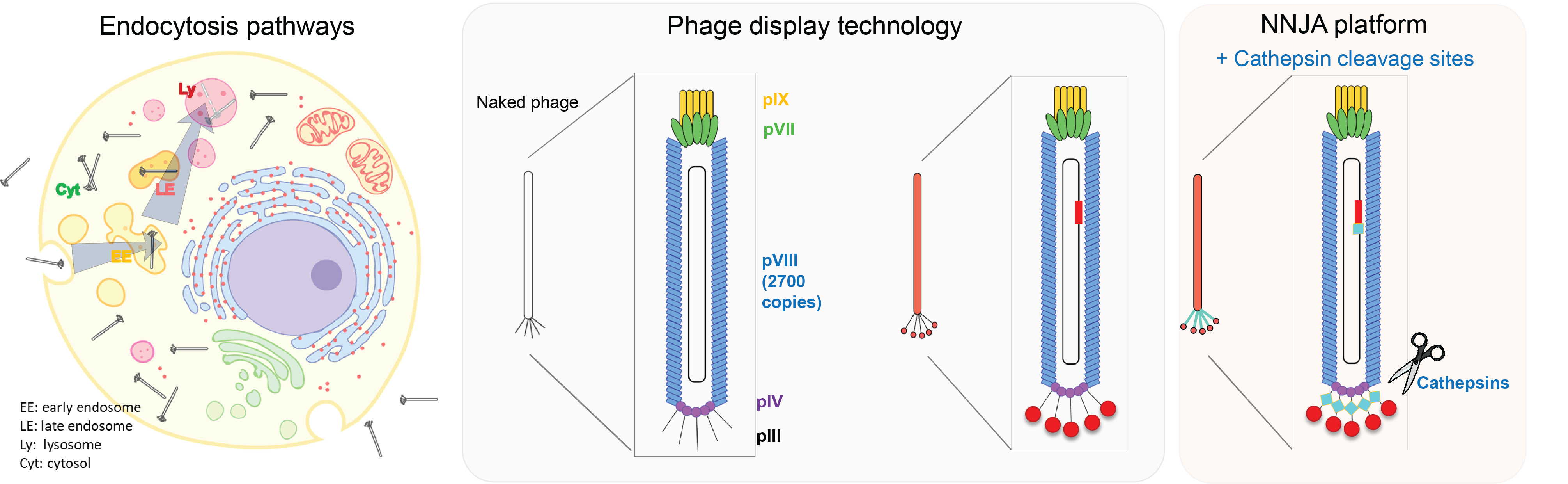


**Supplementary Figure S1.** Schematics of endocytosis pathways, phage display technology and NNJA platform design. Five coat proteins of M13 phage are labeled. Minor coat protein PIII is commonly used for display purpose for peptides, Fabs and other proteins. Gene of interest (encoding region shown in red) can be inserted into phage genome to express corresponding amino acids on the PIII of phage for screening against target of interest. In NNJA platform, in addition to the amino acid sequences displayed on PIII, an engineered substrate sequence recognized and cleaved by mammalian cathepsins was inserted in the GS linker of phage PIII (as shown in diamond shape in cyan color). NNJA, Novel peptides for intracellular delivery by hijacking two cell systems.


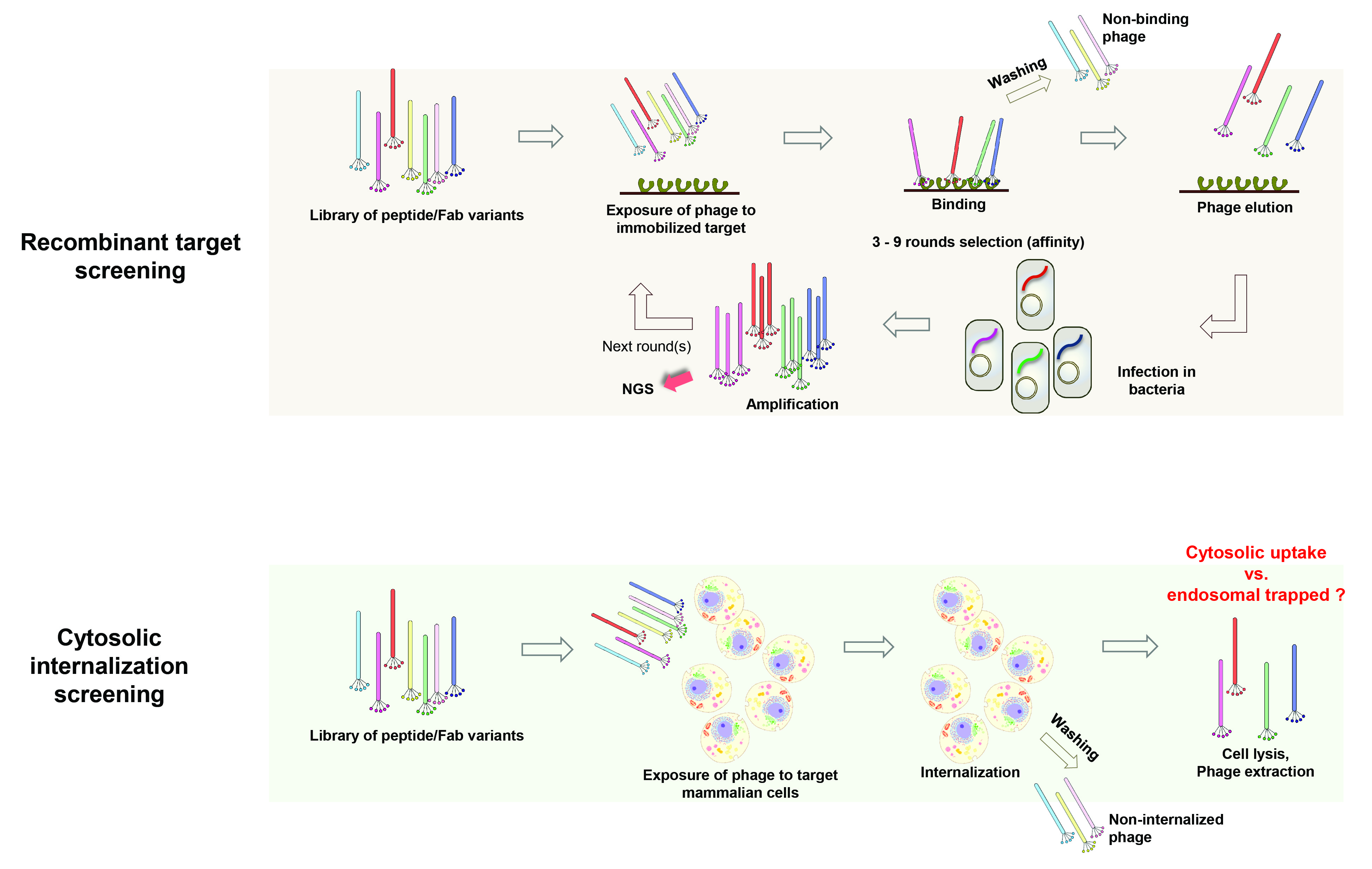


**Supplementary Figure S2**. Schematics of library panning process using Phage display against recombinant targets (upper panel) and main challenges of library selection for cytosolic internalization (lower panel).


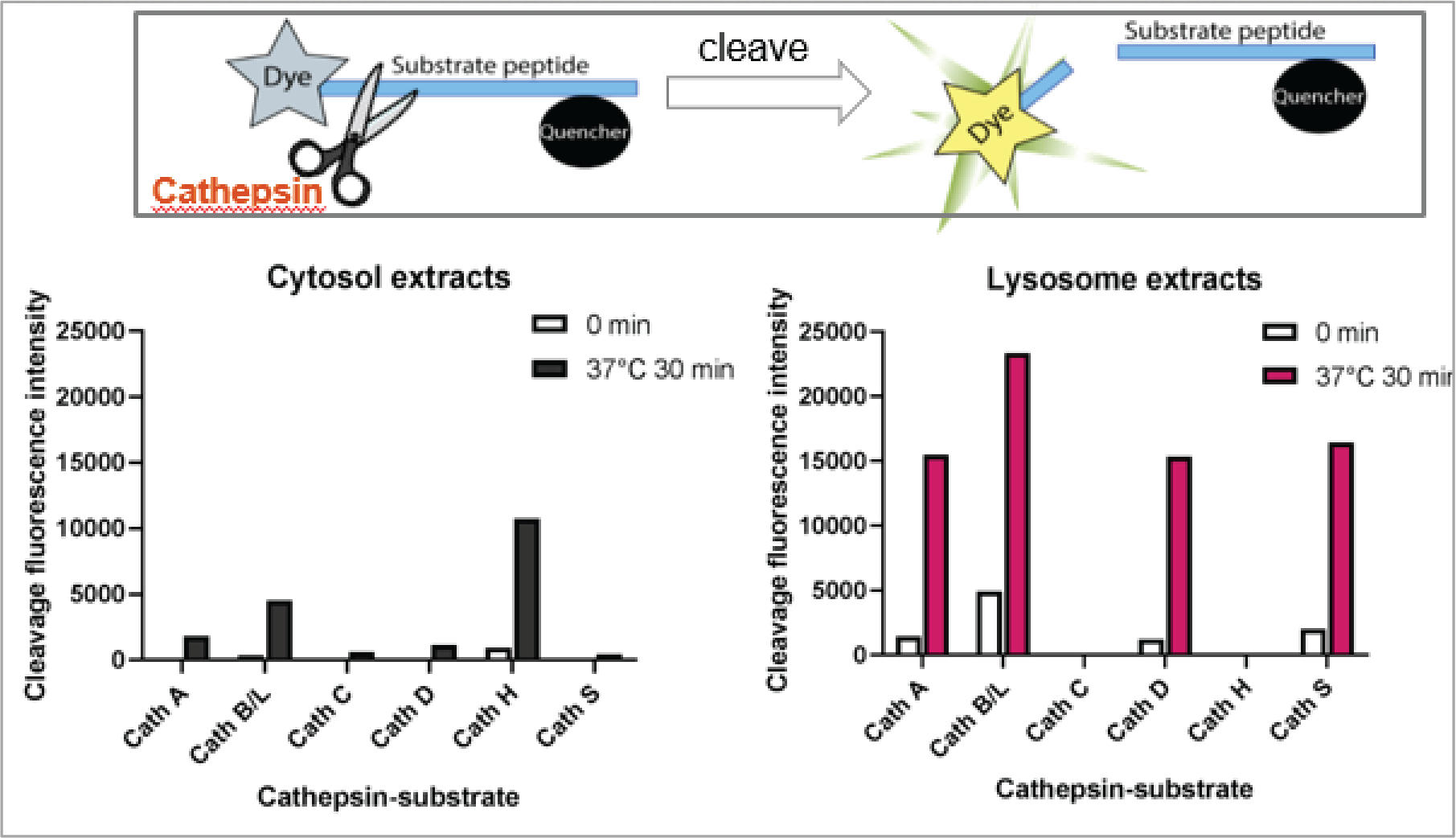


**Supplementary Figure S3**. Cathepsins activities were assessed in cytosol exacts and lysosome extracts using the fluorogenic peptide-substrate assay. Overall low activities were observed after incubation with cytosol extracts excepted for Cathepsin H, the optimal pH of which was reported to be at around 6. Cathepsins A, B/L, D and S showed significant higher activities of substrate cleaving in lysosome extracts under pH 5.


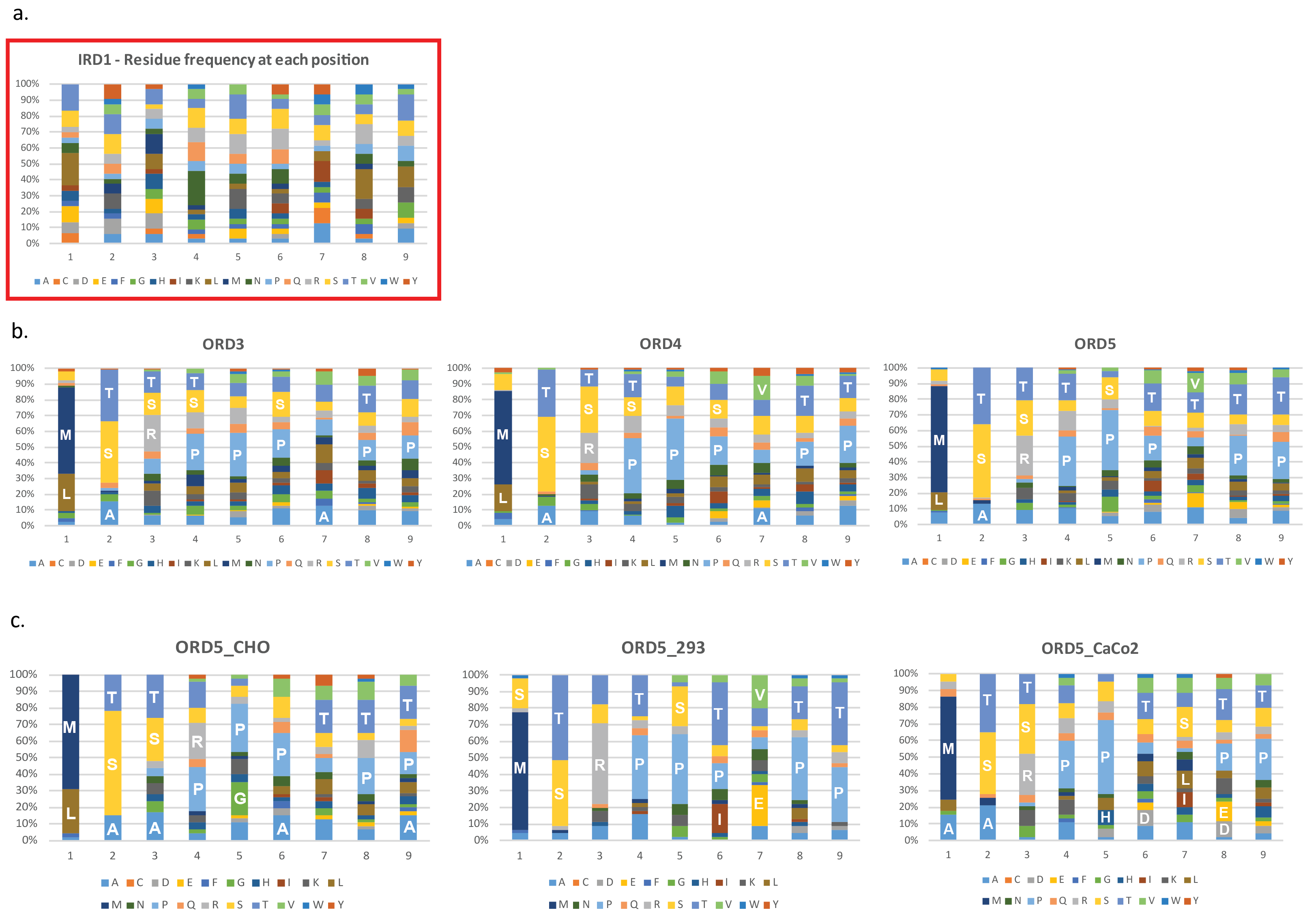


**Supplementary Figure S4**. Amino acids enrichment at each position of 9 residue-sequences from naïve library (**a**), combined sequences of 3 cell types from round 3, 4 and 5 (**b**), and sequences from round 5 of CHO, HEK293 and Caco2 cells (**c**). Each of 20 natural amino acids were coded by colors. The y-axis is the percentage of frequency, and the area of each color block represents how frequent a particular amino acid was shown at that position. The Position 1 is the N-terminus, and position 9 is the C-terminus of a peptide. ORD, output-round; input-round; A, alanine; C, cysteine; D, aspartic acid; E, glutamic acid; F, phenylalanine; G, glycine; H, histidine; I, isoleucine; K, lysine; L, leucine; M, methionine; N, asparagine; P, proline; Q, glutamine; R, arginine; S, serine; T, threonine; V, valine; W, tryptophan; Y, tyrosine.

**
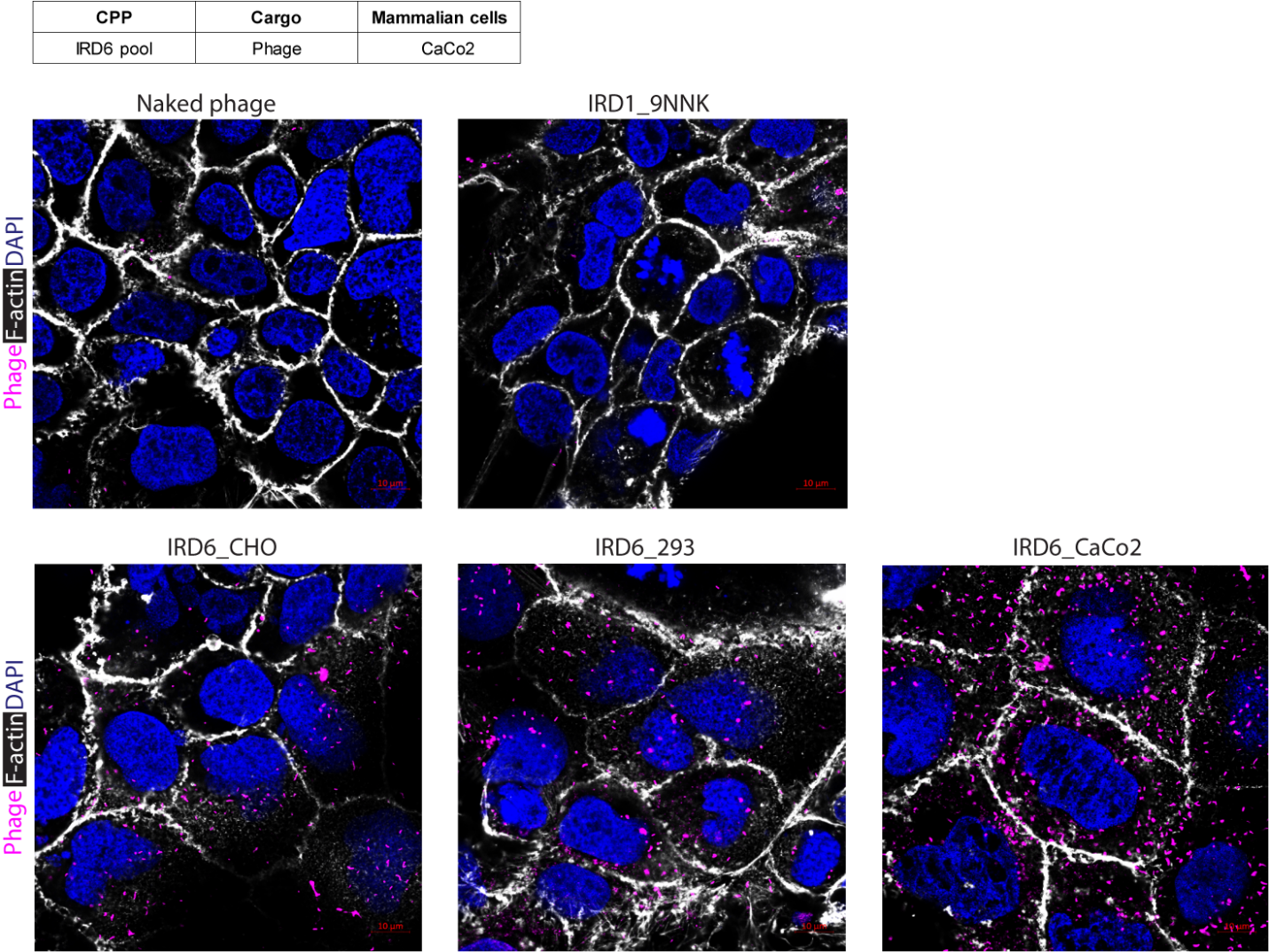
**

**Supplementary Figure S5. Peptide pools in the context of phage selected against three cell types internalized CaCo2 cells by confocal microscopy.**

**Supplementary Table S1. The 37 NNJA peptides selected from NGS.**

|  |  |  |  |  |  |  |  |  |  | **NGS** | | | | | | |
| --- | --- | --- | --- | --- | --- | --- | --- | --- | --- | --- | --- | --- | --- | --- | --- | --- |
|  | **Sequence** | | | | | | | | | **IRD6_CHO** | | **IRD6_293** | | | **IRD6_CaCo2** | |
| **ID** | **1** | **2** | **3** | **4** | **5** | **6** | **7** | **8** | **9** | **Count** | **Frequency** | **Count** | **Frequency** | **Count** | | **Frequency** |
| **NNJA_1** | M | S | T | R | G | P | T | P | A | 27560 | 1.76E-01 | 18047 | 1.42E-01 | 28 | | 2.57E-04 |
| **NNJA_2** | M | T | A | P | A | P | G | L | Q | 17810 | 1.14E-01 | 8045 | 6.33E-02 | 19 | | 1.75E-04 |
| **NNJA_3** | M | T | S | S | S | D | L | R | L | 11345 | 7.26E-02 | 236 | 1.86E-03 | 7 | | 6.429 E-5 |
| **NNJA_4** | L | S | S | R | T | T | Y | Q | G | 4987 | 3.19E-02 | 217 | 1.71E-03 | 52 | | 4.78E-04 |
| **NNJA_5** | M | T | S | K | N | T | Q | I | G | 4278 | 2.74E-02 | 9349 | 7.36E-02 | 5183 | | 4.76E-02 |
| **NNJA_6** | M | S | H | V | G | F | E | T | T | 3968 | 2.54E-02 | 2 | 1.57E-05 | 3 | | 2.76E-05 |
| **NNJA_7** | M | Q | P | M | G | S | T | A | S | 2616 | 1.68E-02 | 60 | 4.72E-04 | 5539 | | 5.09E-02 |
| **NNJA_8** | M | T | P | S | R | L | P | P | S | 2434 | 1.56E-02 | 0 | 0 | 3 | | 2.76E-05 |
| **NNJA_9** | M | S | K | Q | N | Y | H | V | V | 2289 | 1.47E-02 | 1 | 7.87E-06 | 1 | | 9.18E-06 |
| **NNJA_10** | M | A | G | Y | R | S | A | V | N | 1876 | 1.20E-02 | 2 | 1.57E-05 | 1 | | 9.18E-06 |
| **NNJA_11** | M | T | T | K | H | V | A | T | Q | 1615 | 1.03E-02 | 244 | 1.92E-03 | 7957 | | 7.31E-02 |
| **NNJA_12** | M | T | R | T | S | T | E | P | T | 1408 | 9.01E-03 | 16739 | 1.32E-01 | 15 | | 1.38E-04 |
| **NNJA_13** | M | T | T | P | N | P | K | V | R | 1182 | 7.57E-03 | 4501 | 3.54E-02 | 18 | | 1.65E-04 |
| **NNJA_14** | L | T | R | Q | T | N | L | E | V | 1177 | 7.53E-03 | 6 | 4.72E-05 | 3 | | 2.76E-05 |
| **NNJA_15** | S | S | R | P | P | I | V | T | P | 33 | 2.11E-04 | 29342 | 2.31E-01 | 20 | | 1.84E-04 |
| **NNJA_16** | Y | T | R | P | M | S | A | P | N | 94 | 6.02E-04 | 1922 | 1.51E-02 | 3 | | 2.76E-05 |
| **NNJA_17** | F | T | S | P | P | T | E | P | R | 2 | 1.28E-05 | 1647 | 1.30E-02 | 1 | | 9.18E-06 |
| **NNJA_18** | M | G | N | W | T | P | H | G | T | 885 | 5.67E-03 | 4 | 3.15E-05 | 11235 | | 1.03E-01 |
| **NNJA_19** | M | T | S | S | R | D | A | P | A | 3 | 1.92E-05 | 294 | 2.31E-03 | 5306 | | 4.87E-02 |
| **NNJA_20** | M | S | R | Q | S | V | H | T | T | 7 | 4.48E-05 | 24 | 1.89E-04 | 3538 | | 3.25E-02 |
| **NNJA_21** | F | T | S | Q | T | K | V | A | M | 11107 | 7.11E-02 | 1605 | 1.26E-02 | 9 | | 8.27E-05 |
| **NNJA_22** | M | S | R | P | S | S | T | L | L | 2634 | 1.69E-02 | 2 | 1.57E-05 | 4 | | 3.67E-05 |
| **NNJA_23** | M | S | T | P | L | D | R | T | N | 1450 | 9.28E-03 | 0 | 0 | 2 | | 1.84E-05 |
| **NNJA_24** | M | Q | M | A | T | S | T | P | A | 1009 | 6.46E-03 | 1 | 7.87E-06 | 0 | | 0 |
| **NNJA_25** | M | S | K | P | T | R | L | P | V | 31 | 1.98E-04 | 1548 | 1.22E-02 | 1 | | 9.18E-06 |
| **NNJA_26** | L | T | T | T | R | S | L | P | S | 2 | 1.28E-05 | 1176 | 9.25E-03 | 0 | | 0 |
| **NNJA_27** | M | G | S | P | P | T | Y | R | P | 1 | 6.40E-06 | 64 | 5.04E-04 | 2872 | | 2.64E-02 |
| **NNJA_28** | M | S | L | K | S | T | P | H | P | 1 | 6.40E-06 | 23 | 1.81E-04 | 2248 | | 2.07E-02 |
| **NNJA_29** | M | S | T | A | P | P | S | R | T | 0 | 0 | 0 | 0 | 1483 | | 1.36E-02 |
| **NNJA_30** | M | T | S | P | N | I | A | E | P | 1 | 6.40E-06 | 0 | 0 | 1228 | | 1.13E-02 |
| **NNJA_31** | A | S | K | V | P | P | S | G | P | 745 | 4.77E-03 | 428 | 3.37E-03 |  | |  |
| **NNJA_32** | A | A | S | T | R | P | P | Q | L |  |  |  |  | 294 | | 2.70E-03 |
| **NNJA_33** | M | S | Q | R | L | S | H | H | D |  |  |  |  | 673 | | 6.18E-03 |
| **NNJA_34** | R | L | A | K | A | P | P | V | S |  |  |  |  | 249 | | 2.29E-03 |
| **NNJA_35** | M | S | R | T | N | T | T | V | N |  |  |  |  | 623 | | 5.72E-03 |
| **NNJA_36** | M | S | N | P | L | S | L | P | A |  |  |  |  | 172 | | 1.58E-03 |
| **NNJA_37** | M | S | N | T | F | H | R | S | E |  |  |  |  | 281 | | 2.58E-03 |

**
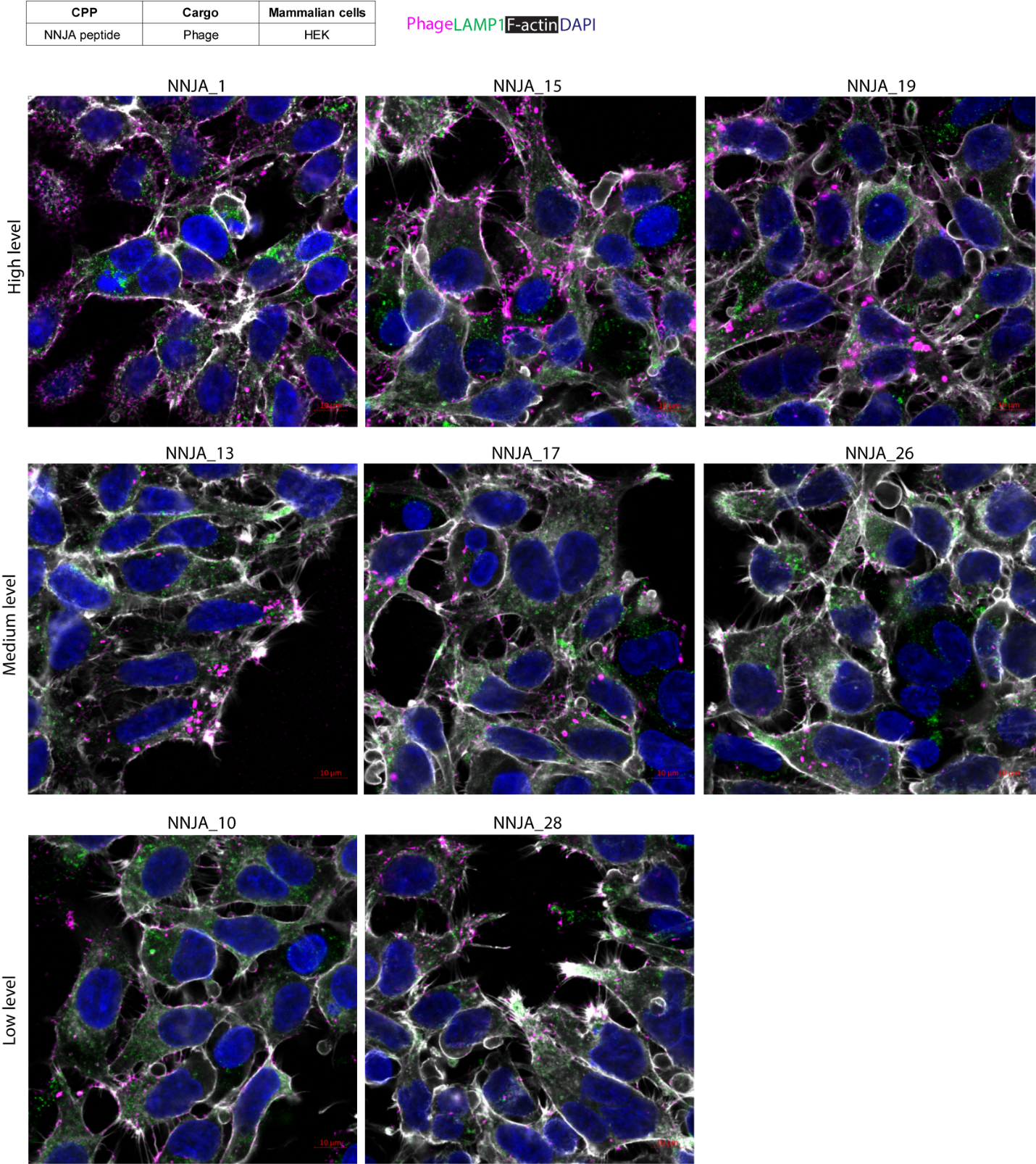
**

**Supplementary Figure S6. Representative images of different internalization levels lead by NNJA peptides caring phage into HEK cells.**

**
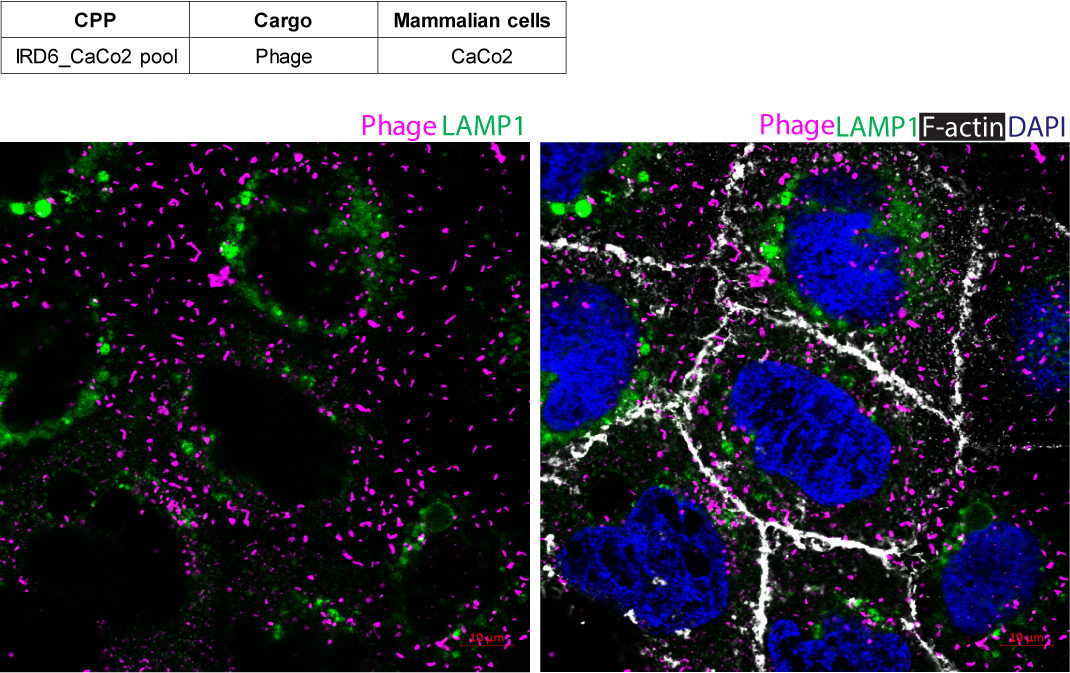
**

**Supplementary Figure S7. Peptide pool selected against CaCo2 cells internalized in CaCo2 cells by confocal microscopy.**

**
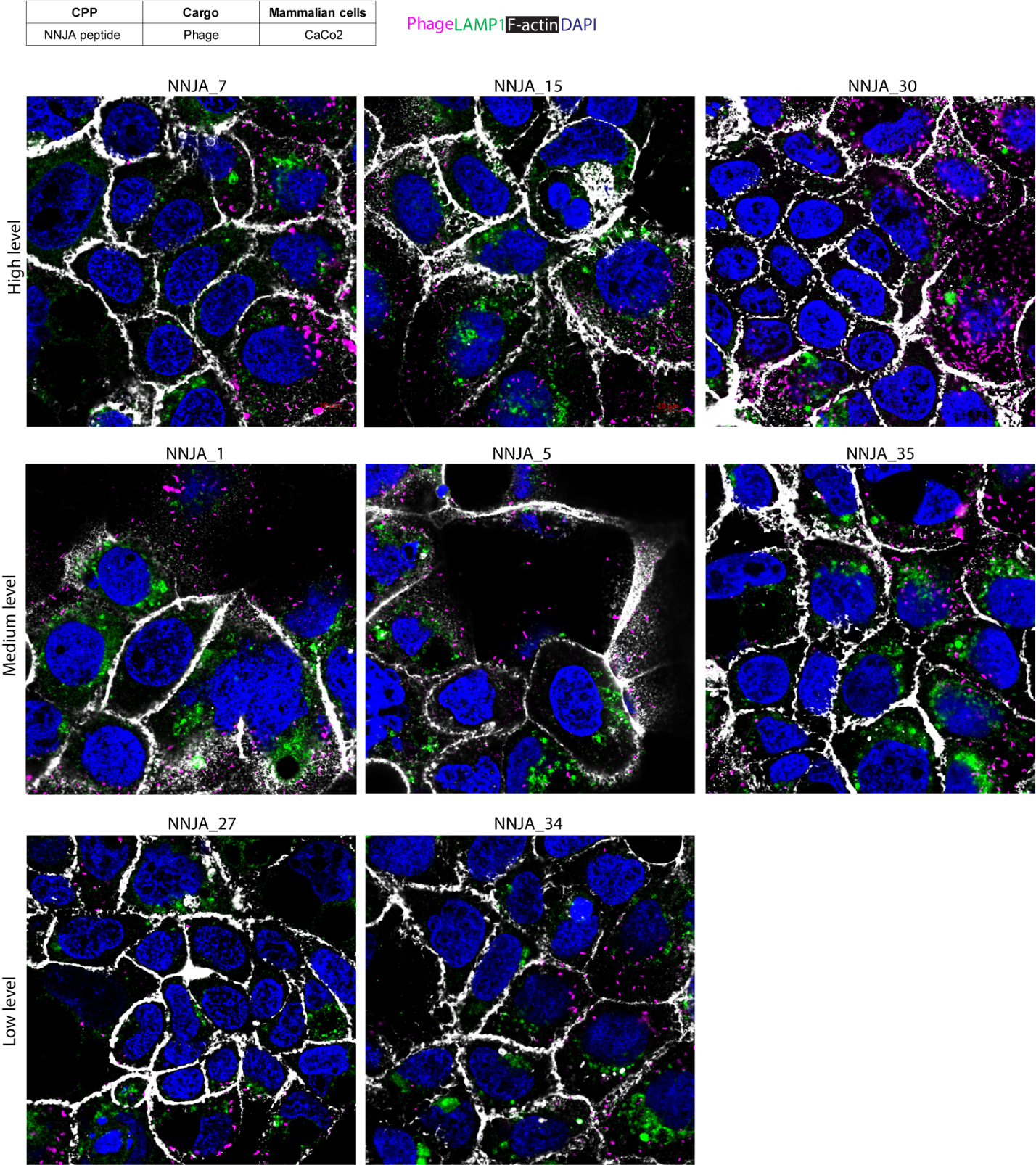
**

**Supplementary Figure S8. Representative images of NNJA-phage internalization in CaCo2 cells.**

**
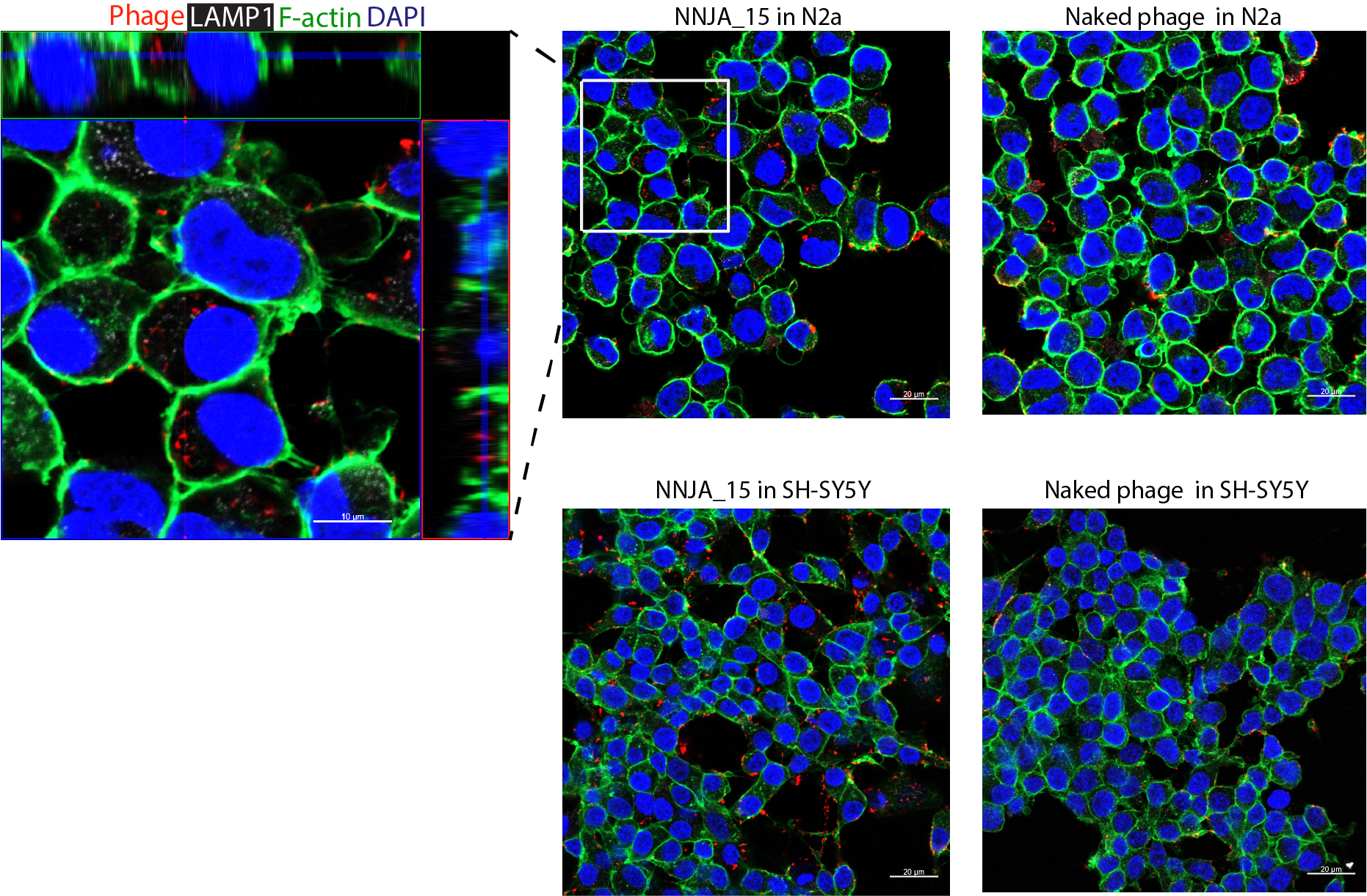
**

**Supplementary Figure S9.** The penetration of NNJA_15 in the context of phage was evaluated in N2a and SHSY5Y cells. Confocal imaging confirmed the localization of NNJA_15-phage in cytoplasmic domain.


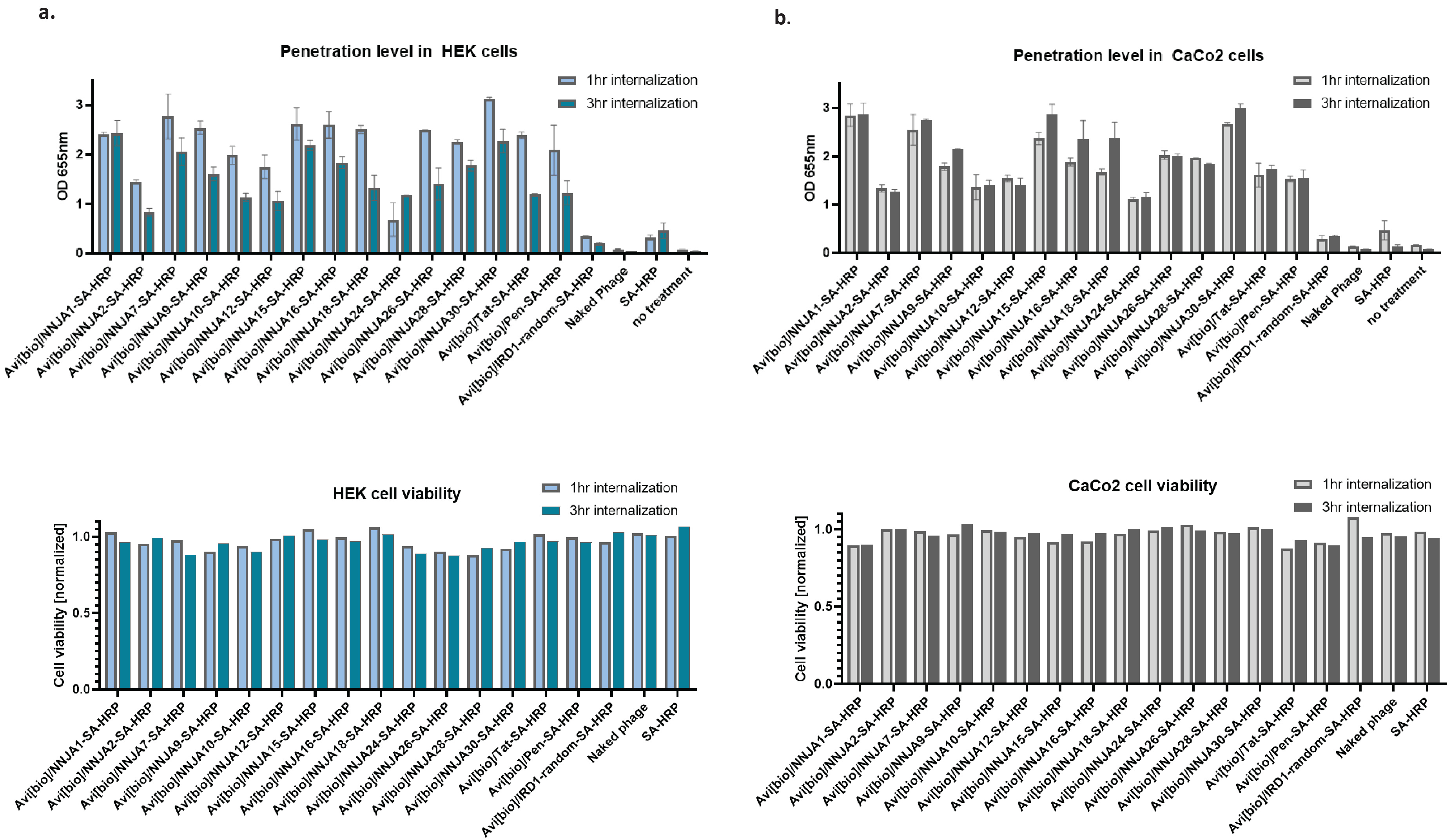


**Supplementary Figure S10. Penetration levels of the selected phage-protein complex in cells.** Thirteen NNJA peptides together with Tat and penetratin were displayed on homogenous phage clones in addition to the Avi-tag on pIX. After binding to SA-HRP, the phage-protein complex was subjected to cells for internalization for 1 hr or 3 hr in HEK cells **(a)** and Caco2 cells **(b)**. The viability of cells was evaluated accordingly.


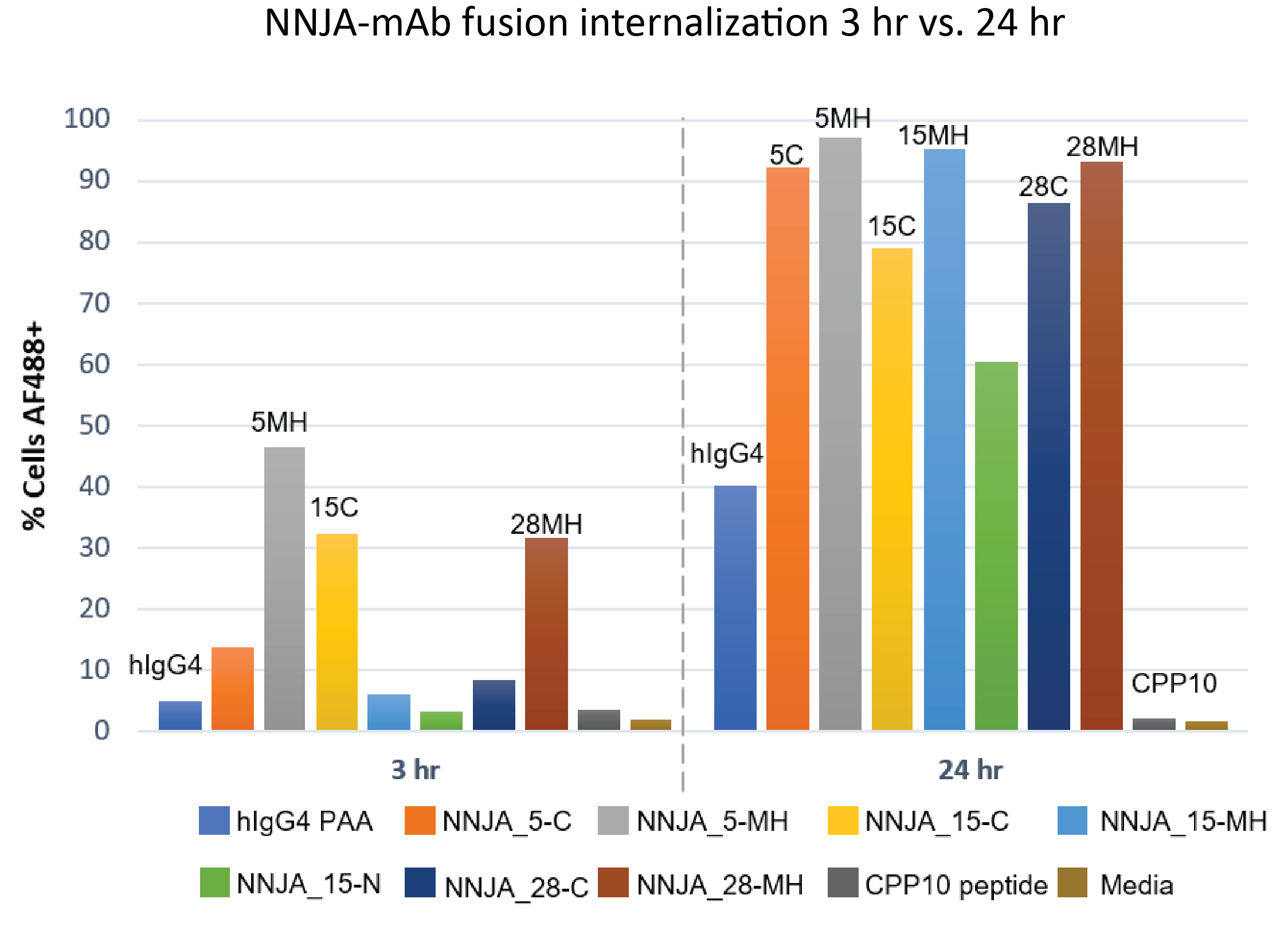


**Supplementary Figure S11**. The internalization level of NNJA-mAb fusion in SH-SY5Y cells after 3 hr and 24 hr by flow cytometry. Selected NNJA peptides (NNJA_5, 15 and 28) were inserted into various locations including minor hinge (MH), N-terminus (Nterm) or C-terminus (Cterm) of heavy chain of isotype control IgG4 antibody by cloning and expression. Alexa fluorophore 488 was site-directed conjugated to the engineered Cysteine residue in CH1 region. The internalization levels were detected by flow cytometry and the percentage of positive cell population was presented at y-axis. NNJA_5-MH, NNJA_15-Cterm, NNJA_28-MH showed substrantial uptake at 3 hr compared to the hIgG4 control only. NNJA_5-Cterm, NNJA_15-MH and NNJA_28-Cterm showed substantial uptake until 24 hr time point indicating a different kinetics of internalization. CPP10-AF488 peptide (no cargo attached) (or CPP-12 as named in the original published study) was used as control, which showed no uptake after 3 hr and 24 hr incubation. No cell death observed from all groups under the condition tested.

**Supplementary Table S2. Peptide counts from NGS by sampling phage from each round selection per cell type.**

| **NGS counts** | **Total sequences after trimming** | **Unique sequences** | **Unique %** |
| --- | --- | --- | --- |
| **naïve library** | 113,186 | 111,662 | 99% |

|  | **293 selection arm** | | | **Caco2 selection arm** | | | **CHO selection arm** | | |
| --- | --- | --- | --- | --- | --- | --- | --- | --- | --- |
| **NGS counts** | **Total after trimming** | **Unique** | **Unique %** | **Total after trimming** | **Unique** | **Unique %** | **Total after trimming** | **Unique** | **Unique %** |
| **Round 2** | 49,853 | 44,412 | 89% | 134,945 | 67,456 | 50% | 140,835 | 123814 | 88% |
| **Round 3** | 209,542 | 78,980 | 38% | 223,334 | 54,470 | 24% | 184,134 | 96456 | 52% |
| **Round 4** | 151,070 | 33,507 | 22% | 124,719 | 27,370 | 22% | 146,484 | 46814 | 32% |
| **Round 5** | 127,076 | 14,871 | 12% | 108,884 | 17,897 | 16% | 156,222 | 24325 | 16% |


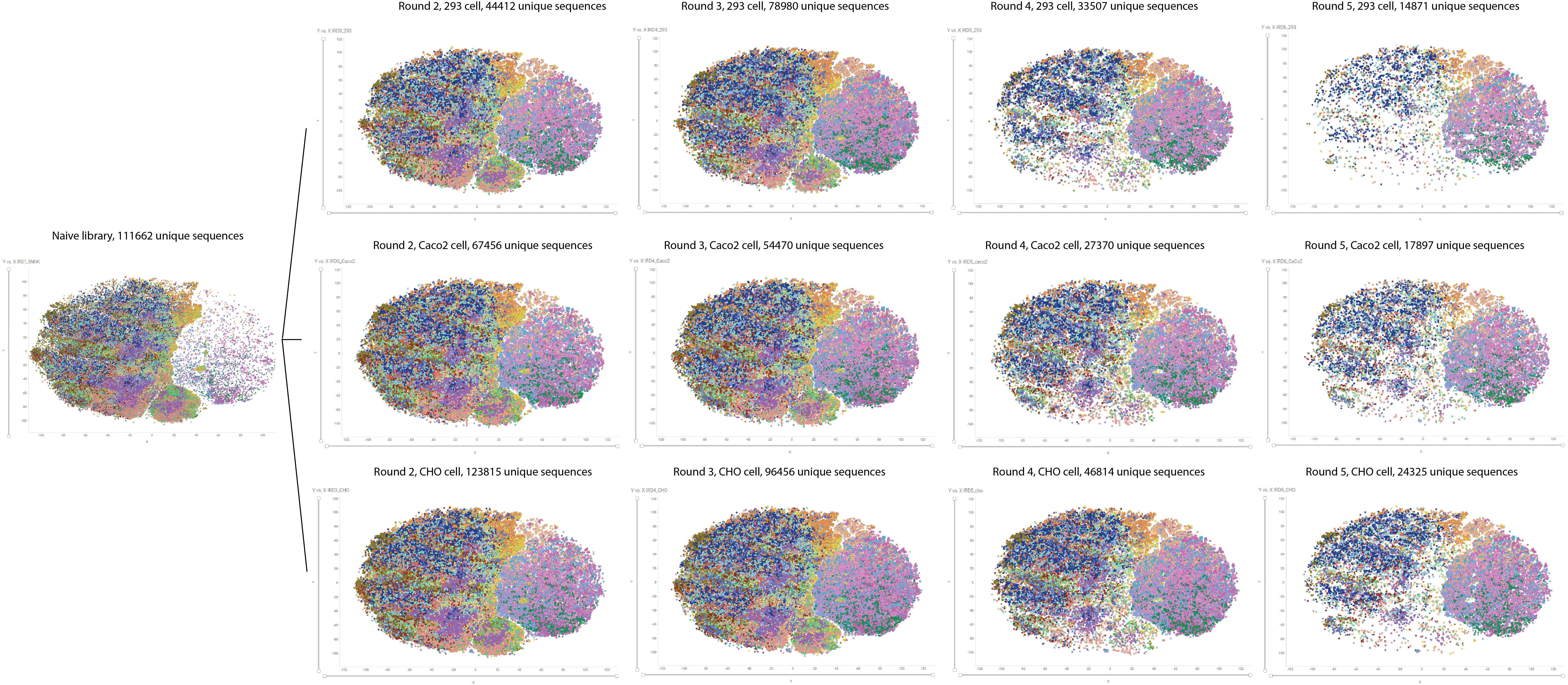


**Supplementary Figure S12. Global clustering analysis with all peptide sequences as in one data set.** NGS counts ≥ 1 are all included in this graph. Evolution of individual peptide is visualized in the map through out selection rounds. Each dot represents a unique peptide. Colors represented the cluster group based on sequence similarity. Size of the dot shows the counts of the peptide from NGS.

**
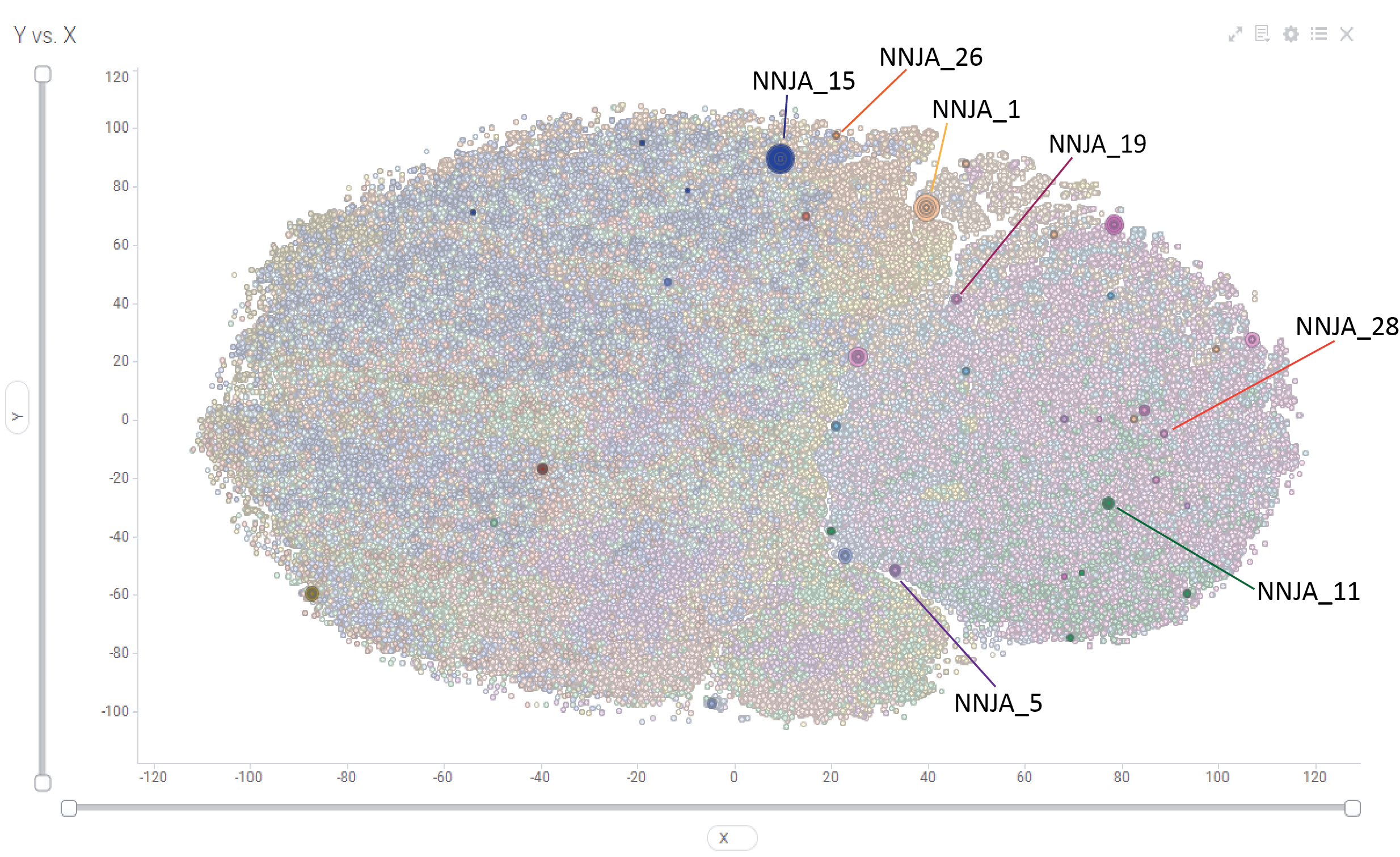
**

**Supplementary Figure S13. The distribution of 37 tested NNJA peptides in the global clustering map.** Among the 37, the 7 NNJA tested as siRNA conjugates were highlighted and labeled. Individual dot represents a unique peptide sequence from naïve library and all three selection arms (13 samples in total). The colors represent 20 clusters generated by sequence similarity analysis, and the size of individual dot indicate the enrichment of peptide after 5 rounds selection. Only the 37 NNJA peptides are highlighted in this figure, with the rest of the sequences in dimmer colors in the background.


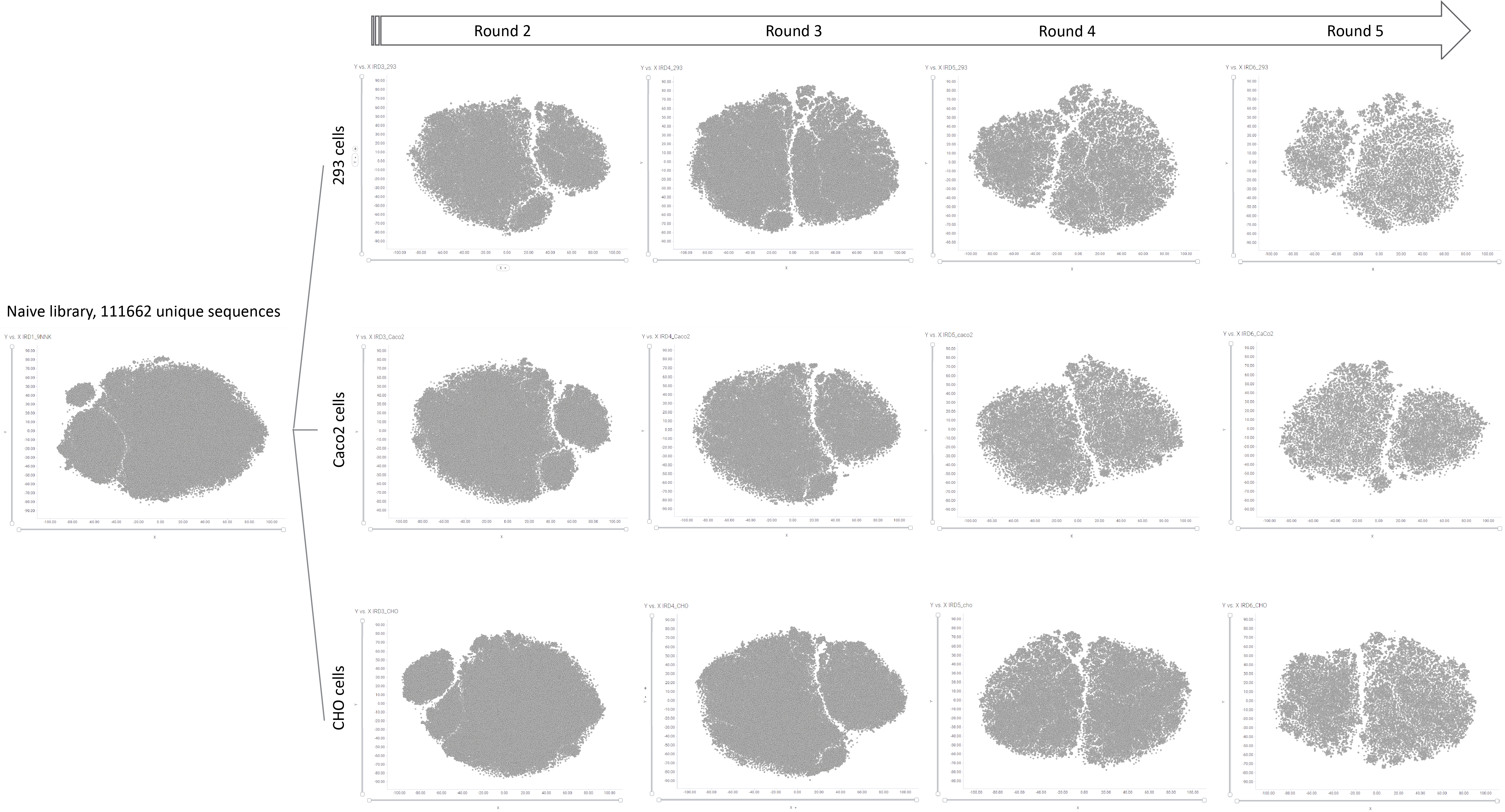


**Supplementary Figure S14.** The High-level evolution of peptide sequences from naïve library throughout cell selection by looking into sequence similarity embedding for individual dataset. The shapes of each island in naïve library map changed dramatically in maps of later rounds selection. The x/y ordinates are relative numbers without actual meaning of values. Each dot represents a unique peptide sequence.


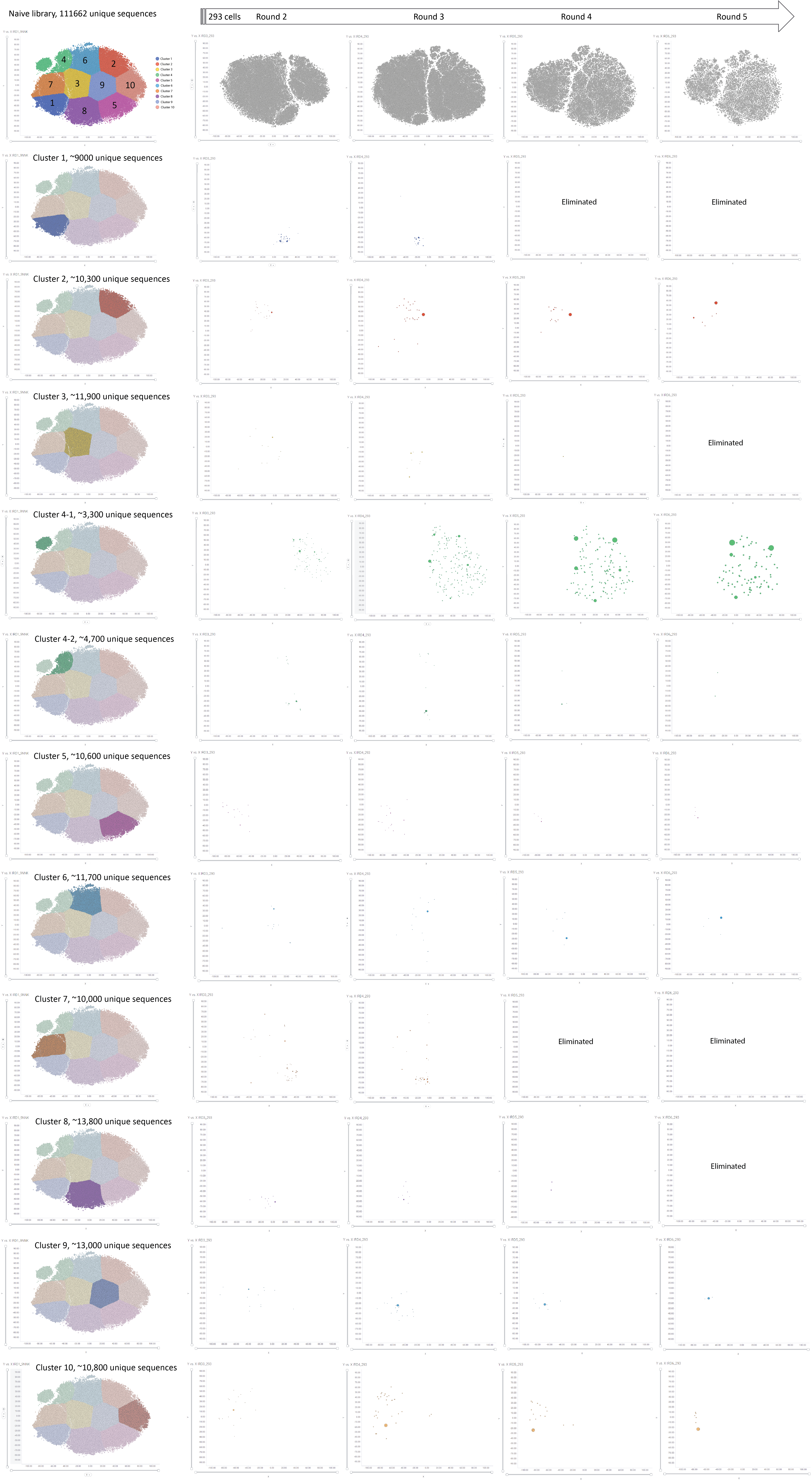


**Supplementary Figure S15**. Evolution of peptides of individual cluster from naïve library and enrichment after 2-5 rounds selection in 293 cells. Sequences from cluster 2 and cluster 4-1 are significantly enriched as cell selection progresses. On the other hand, sequences from cluster 1, 3, 7,8 are completely eliminated after 5 rounds selection.


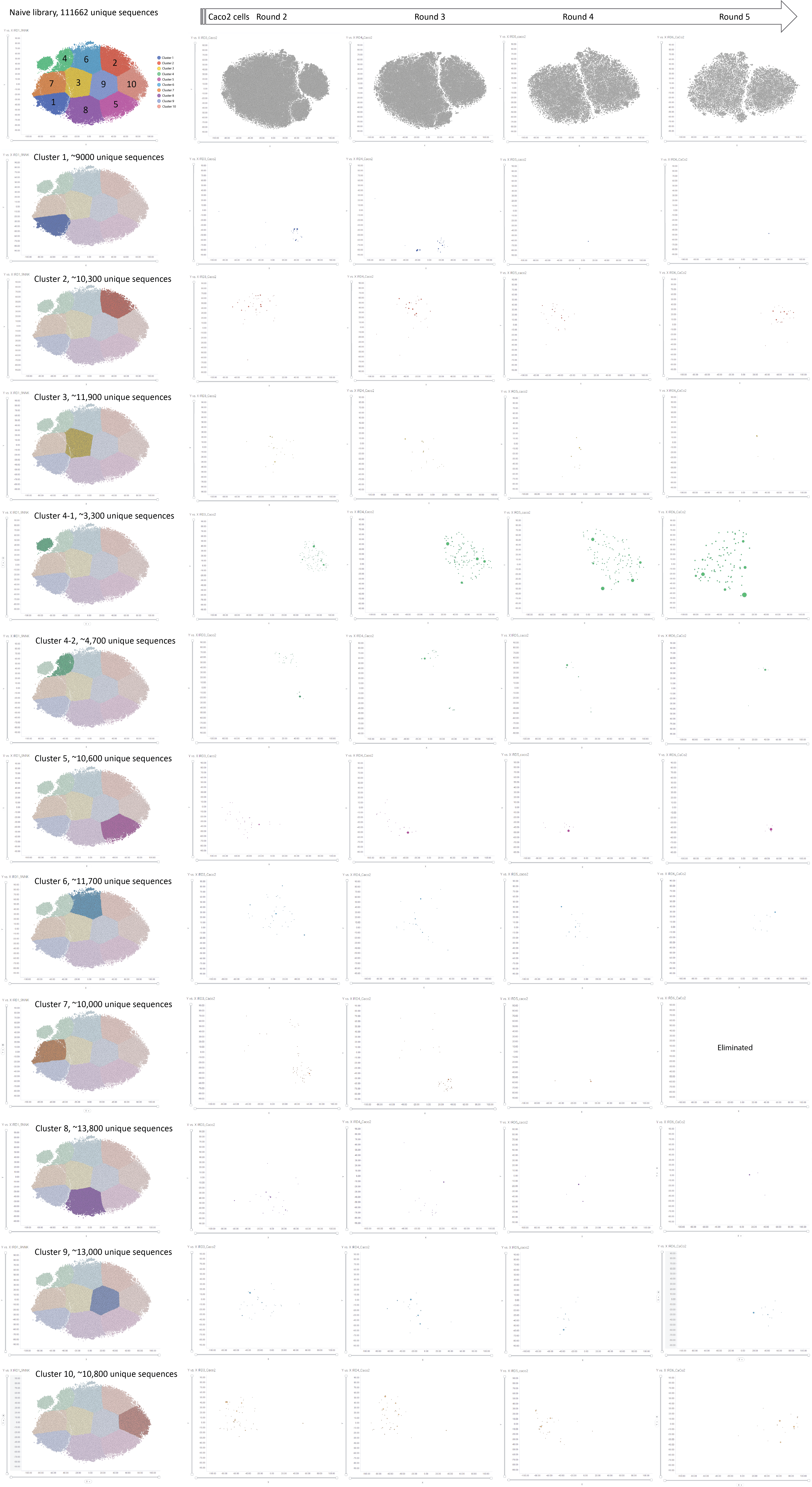


**Supplementary Figure S16**. Evolution of peptides of individual cluster from naïve library and enrichment after 2-5 rounds selection in Caco2 cells. Sequences from cluster 4-1 are significantly enriched as cell selection progresses. Sequences from cluster 9 are modestly enriched, while sequences from cluster 7 are completely eliminated after 5 selection.


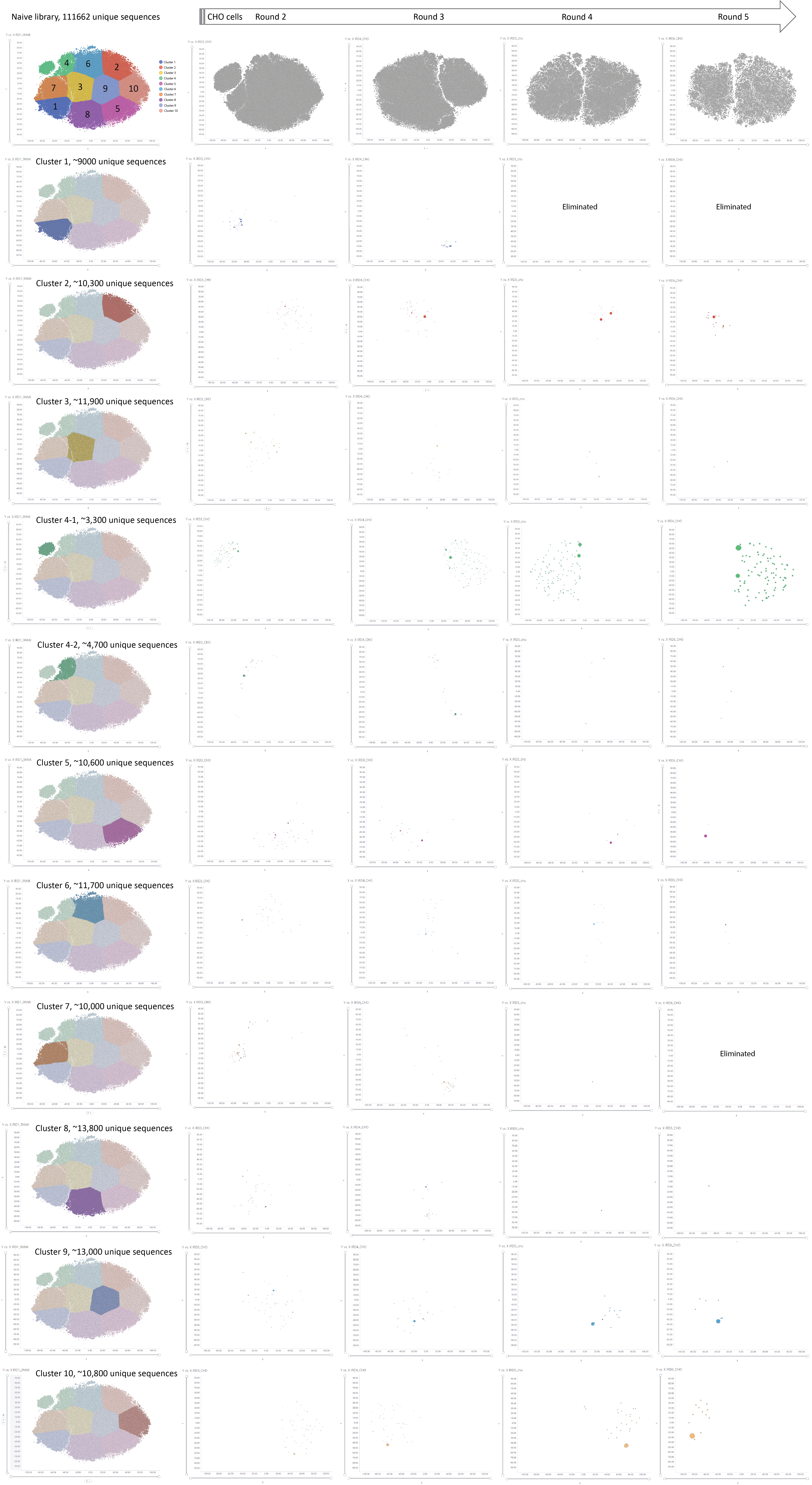


**Supplementary Figure S17**. Evolution of peptides of individual cluster from naïve library and enrichment after 2-5 rounds selection in CHO cells. Sequences from cluster 4-1, 9 and 10 are significantly enriched as cell selection progresses. On the other hand, sequences from cluster 1, 7 are eliminated after 5 rounds selection.


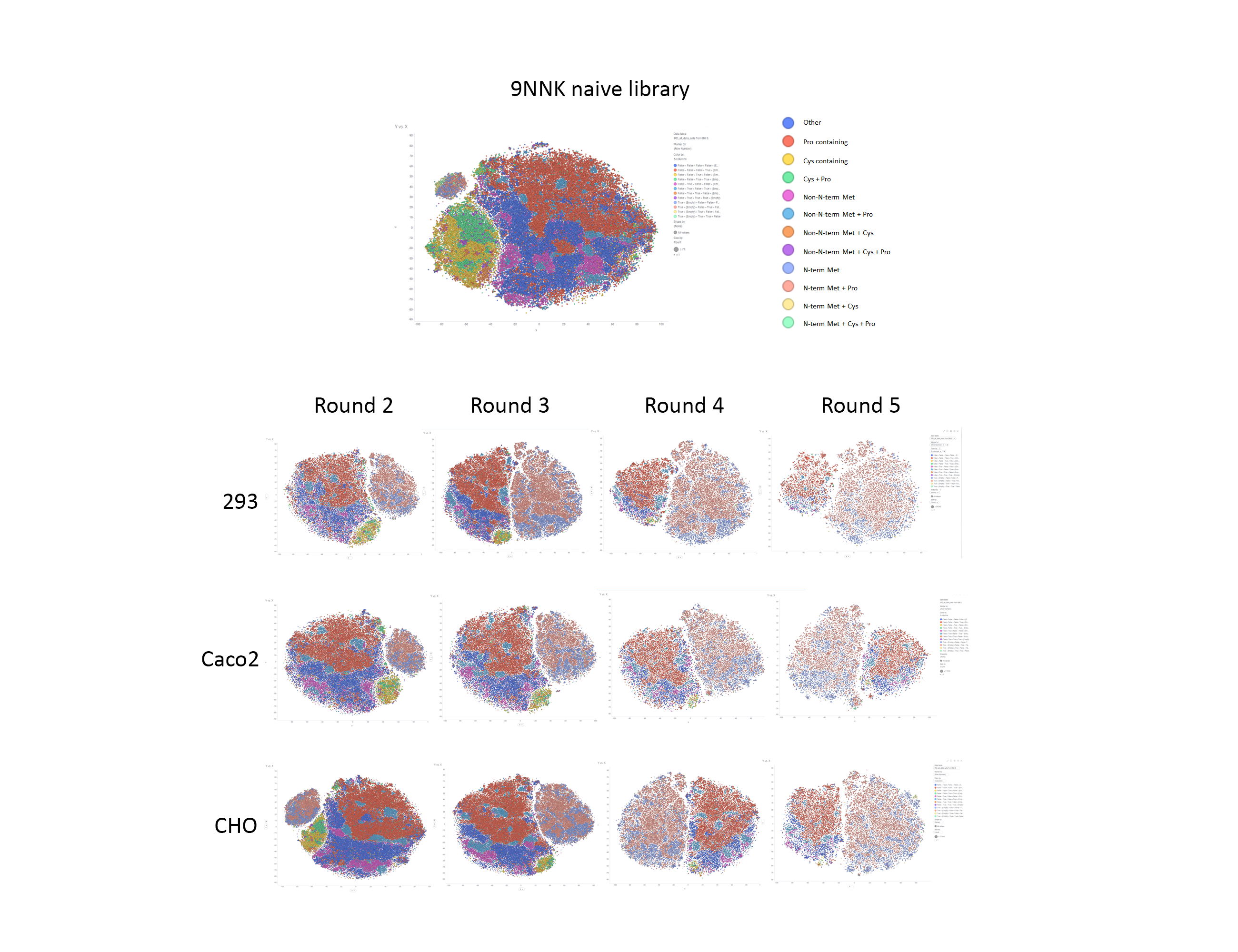


**Supplementary Figure S18**. Additional views of sequence evolution with N-term Methionine, Cysteine and Proline amino acids from naïve library to the later rounds selection.


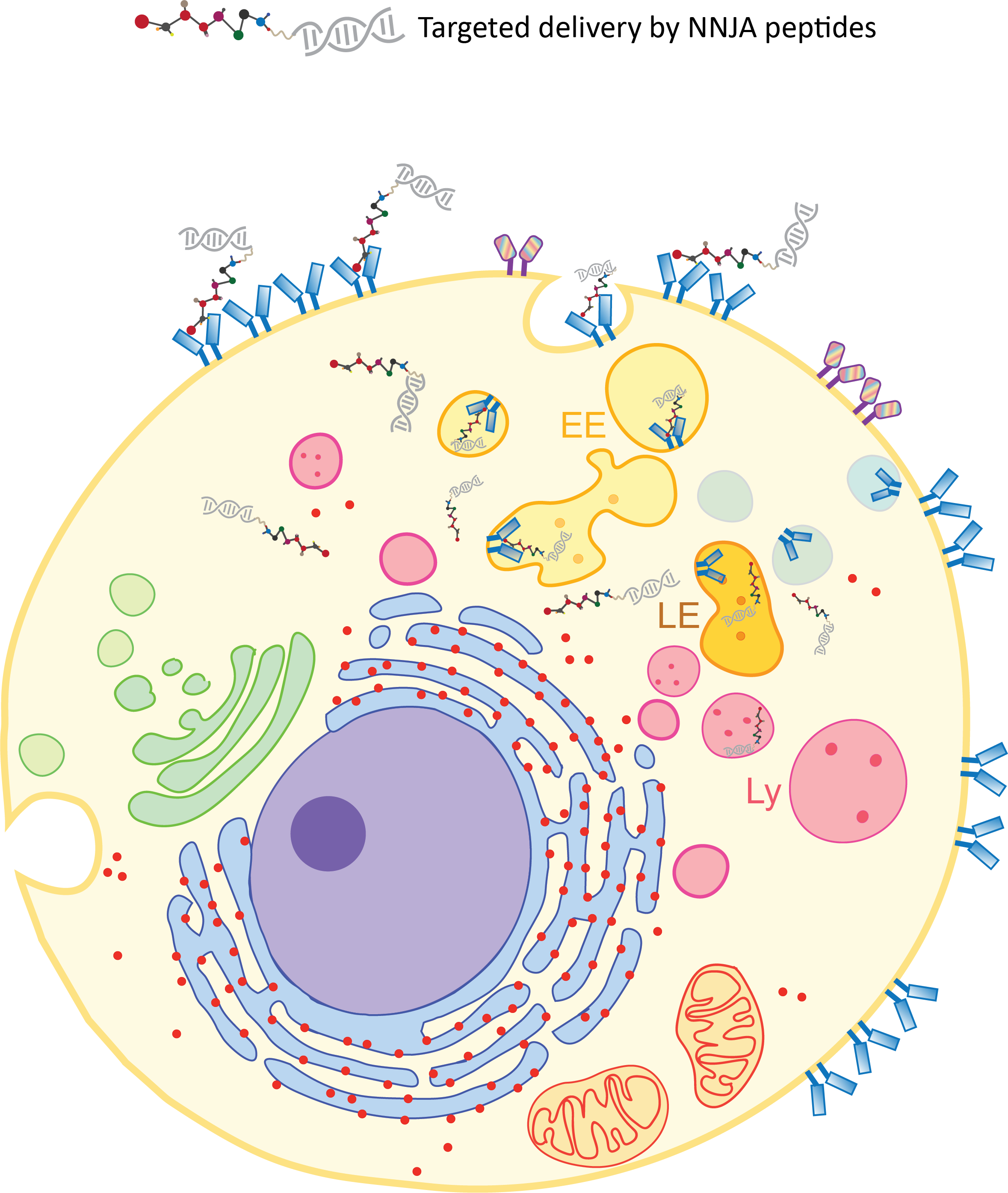


**Supplementary Figure S19**. Schematics of targeted delivery using NNJA platform via receptor-mediated endocytosis to select peptides that escape from endo-lysosomes, and localize inside cytosol. Examples of cargoes are not limited to antibody, peptide, oligonucleotides, proteins.
